# Water-Soluble Inclusion Complexation of Naphthyl-Containing Thiosemicarbazides and Thioureas with β-Cyclodextrin

**DOI:** 10.3390/molecules31081290

**Published:** 2026-04-15

**Authors:** Oralgazy A. Nurkenov, Zainulla M. Muldakhmetov, Serik D. Fazylov, Anel Zh. Mendibayeva, Irina A. Pustolaikina, Akmaral Zh. Sarsenbekova, Olzhas T. Seilkhanov, Ardak K. Syzdykov, Saule K. Kabiyeva, Zhangeldy S. Nurmaganbetov

**Affiliations:** 1 Laboratory of Synthesis of Biologically Active Substances, Institute of Organic Synthesis and Coal Chemistry of the Republic of Kazakhstan, Karaganda 100008, Kazakhstan; nurkenov_oral@mail.ru (O.A.N.); iosu.rk@mail.ru (Z.M.M.);; 2 Department of Chemical Technology and Ecology, Karaganda Industrial University, Temirtau 101400, Kazakhstan; 3 Department of Physical and Analytical Chemistry, Buketov Karaganda National Research University, Karaganda 100024, Kazakhstan; 4 Laboratory of Engineering Profile NMR-Spectroscopy, Sh. Ualikhanov Kokshetau University, Kokshetau 120000, Kazakhstan

**Keywords:** thiosemicarbazides, thioureas, β-cyclodextrin, molecular docking, antiviral activity, plasma viscosity, thermogravimetric analysis

## Abstract

The paper presents the synthesis of new naphthyl-containing derivatives of thiosemicarbazide and thiourea, their water-soluble inclusion complexes with β-cyclodextrin, as well as an assessment of their potential antiviral and hemorheological activity. As a criterion for the specific antiviral effect of new compounds, their chemotherapeutic indices were calculated using predictive analytics tools driven by artificial intelligence and molecular docking methods. Molecular docking studies with three protein targets PknB (2FUM), DprE1 (6HEZ), and InhA (1ENY) confirmed strong and specific ligand–protein interactions. The effects of structural features of new compounds on the rheological characteristics of blood were considered, and the most promising samples were identified for further in-depth in vitro study of their specific biological activity. The performed thermoanalytical study showed that the structure of the included ligand, as well as the shape of the receptor, significantly affect the thermal stability and kinetic parameters of the decomposition of the inclusion complex. In silico evaluation of the newly synthesized compounds revealed promising biological activity profiles, with all compounds demonstrating predicted antimycobacterial and antituberculosis potential. In silico analysis of the newly synthesized compounds revealed favorable biological activity profiles, with all candidates demonstrating predicted antimycobacterial and antituberculosis potential.

## 1. Introduction

The increasing frequency of severe viral infections, particularly in the wake of the COVID-19 pandemic, underscores the urgent need for more effective antiviral therapeutics. Coronavirus infection (COVID-19) is caused by a new virus known as SARS-CoV-2 severe acute respiratory syndrome coronavirus. Hyperinflammation caused by SARS-CoV-2, as well as changes in plasma proteins, red blood cell deformability, and platelet activation affect blood viscosity [1]. Hyperviscosity syndrome develops in various hematological diseases, including multiple myeloma, sickle cell anemia, Waldenstrom’s macroglobulinemia, polycythemia, and leukemia [2]. High blood viscosity in influenza pneumonia and respiratory viral infections can provoke the progression of thrombosis due to increased vascular resistance, which makes perfusion of peripheral tissues difficult. Increased blood viscosity contributes to the development of myocardial infarction (MI), venous thrombosis, and venous thromboembolism in acute infections [3]. Consequently, a clear correlation exists between SARS-CoV-2 infection and alterations in blood viscosity in patients with COVID-19 [4]. Thus, these observations indicate that acute respiratory viral infections, as well as other viral infections, may increase the risk of vascular complications due to the induction and progression of viral infections.

Currently, various classes of antiviral drugs are known that contain a diaryl ring in their structure [5,6] and have pronounced inhibitory activity in vitro against HIV and other viruses. As a rule, aromatic residues in their structure are associated with a heterocyclic component, represented in many cases by a nitrogenous base, through a nitrogen atom of the latter. However, in these compounds, the influence of structural factors on the nature of their antiviral activity has not been studied in sufficient detail. In addition, the antiviral potential of heterocyclic structures containing the naphthalene fragment is illustrated by only a small number of examples. The simplest representatives of antiviral compounds containing naphthalene core are “bonaphthon” and “oxolin”. Compounds based on thiosemicarbazides and naphthyl sulfonamides [7,8] are of particular scientific and practical value among organosulfur derivatives, which are widely used as components of antimicrobial [9,10], antiviral [11], antituberculous, and anti-HIV-1 and HIV-2 agents [12,13]. These substances are also widely used as a platform for the development of new hemorheological drugs that exhibit their effect in micro- and nanomolar concentrations [14,15,16]. However, despite the achievements in the field of studying the molecular mechanisms of hemorheological disorders, the arsenal of effective pharmacological agents capable of correcting these changes remains extremely limited.

This paper presents the results of a study of the structure, molecular docking, antiviral and hemorheological activity of new naphthyl thiosemicarbazides and naphthyl thioureas. The synthesized naphthyl-containing thiosemicarbazides and thioureas have low solubility in aqueous media, which complicates a comprehensive assessment of their biological properties. In this regard, they were converted into water-soluble inclusion complexes with β-cyclodextrin (β-CD), which acts as the host molecule. The study and establishment of the relationship between the structural and thermodynamic characteristics of the inclusion complexes of naphthyl-containing thiosemicarbazides and thioureas with β-cyclodextrin, considered in the work, have not been carried out previously. To date, the relationship between the structural and thermodynamic features of β-cyclodextrin inclusion complexes with naphthyl-containing thiosemicarbazides and thioureas remains unexplored.

## 2. Results and Discussion

### 2.1. Description of Syntheses and Characteristics of New Naphthyl-Containing Thiosemicarbazides, Naphthyl Thioureas and Their Inclusion Complexes with β-Cyclodextrin

**Figure 1:**
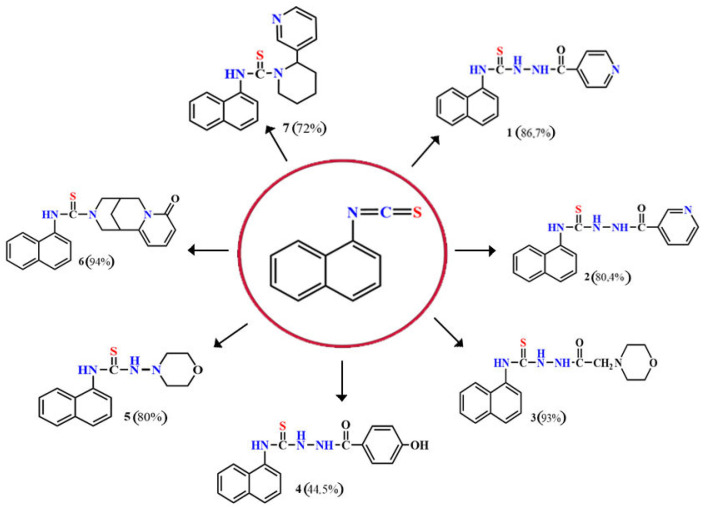
New naphthyl thiosemicarbazides and naphthyl thioureas.

Naphthyl-containing compounds were the objects of study (Figure 1) [17]:

N-(naphthalene-1-yl)-2-isonicothionyl-hydrazino-1carbothioamide **1**,

N-(naphthalene-1-yl)-2-nicothionyl-hydrazino-1-carbothioamide **2**,

N-2-4(4-hydroxybenzoyl)-(naphthalene-1-yl)-hydrazino-1-carbothioamide **3**,

1-morpholino-3-(naphthalene-1-yl)-thiourea **4**,

N-2-(2-morpholinoacetyl)-(naphthalene-1-yl)-hydrazino-1-carbothioamide **5**,

1N-(Naphthalene-1-yl)cytisino-1-carbothioamide **6**,

N-(Naphthalene-1-yl)anabasino-1-carbothioamide **7**.

The IR spectra of the synthesized compounds **1**–**7** have absorption bands in the range of 1519–1500 cm^−1^, characteristic of the C=S group of the thiosemicarbazide fragment, and absorption bands of the C(O) amide group NH manifests itself in the region of 1682 cm^−1^, the NH group manifests itself as a peak of average intensity at 3279–3236 cm^−1^ (Figure S1). The data of the new compounds obtained were also confirmed by NMR spectra (Figures S2 and S3).

**Figure 2:**
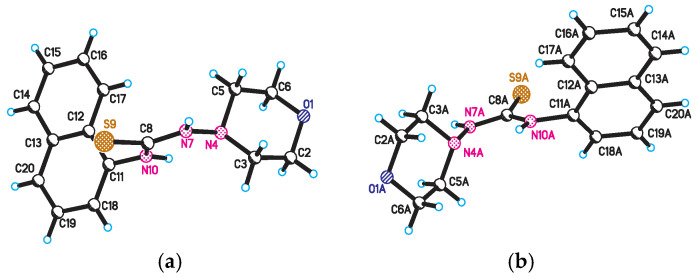
The structure of molecule **4**: (**a**) conformation **4a**; (**b**) conformation **4b**.

X-ray diffraction study of the structure of 1-morpholino-3-(naphthalene-1-yl)thiourea **4** was performed in order to establish the spatial structure of naphthyl-containing thiosemicarbazide molecules. The structure of molecule **4** is shown in Figure 2.

**Figure 3:**
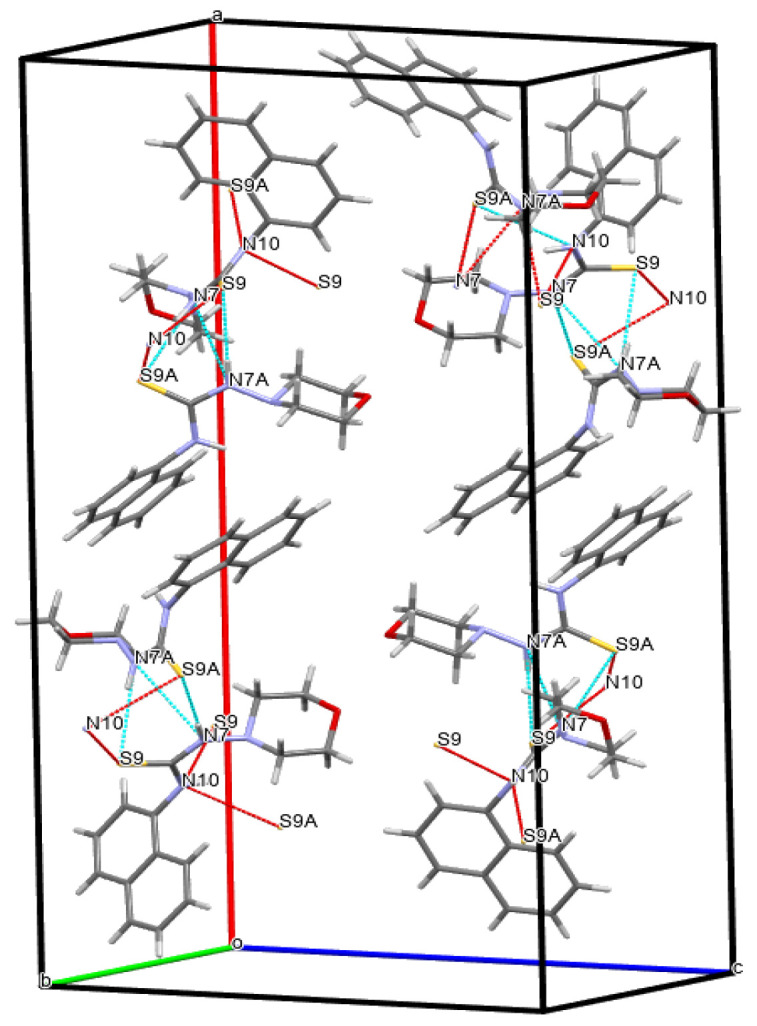
Fragment of the elementary crystal lattice of compound **4**, showing the arrangement of two independent molecules (**4a** and **4b**) and a system of intermolecular hydrogen bonds that stabilize the position of the sulfur atom S9 along the a axis.

The results indicate that the bond lengths and valence angles in compound **4** are consistent with literature data and are characteristic of the obtained compounds: 1-(1-naphthyl)thiourea, N-(naphthalene-1-yl)pyridine-2-carbothioamide, N-naphthalene-1-il-N′-[(pyridine-4-yl)methyl]thiourea [17]. The elementary crystal lattice **4** contains two identical molecules **4a** and **4b**. The geometric parameters **4a** and **4b** are the same except the position of the thioamide fragment relative to the naphthalene frame. In these structures, the torsion angles of the C8-N10-C11-C18 and C8A-N10A-C11A-C18A atoms are 92.91° and −59.83°, respectively, indicating the equatorial orientation of the morpholylthioamide group relative to this framework [18]. The naphthalene framework **4a** and **4b** are flat with an accuracy of 0.006 Å and 0.01 Å, respectively. The position of the sulfur atom S9 is stabilized by the presence of two intermolecular hydrogen bonds S9…H10-N10 (1.5 − x, 1/2 + y,z) (distance N-H 0.86 Å, S…H 3.04 Å, S…N 3.54 Å) and S9…H7-N7(x,1 − y,1/2 + z) (distance N-H 0.86 Å, S…H 2.51 Å, S…N 3.37 Å), which forms a stable compound of two independent molecules along the a axis in the crystal lattice (Figure 3).

The morpholyl cycle in molecules **4a** and **4b** assumes a conformation close to a somewhat distorted chair (asymmetry parameter ΔC_2_^1,6^ = 0.54, ΔC_s_^1^ = 0.17 **4a**, ΔC_2_^1,6^ = 1.00, ΔC_s_^1^ = 1.00 **4b**). The position of the nitrogen atom N7 and N7A relative to the morpholyl cycle is equatorial (the torsion angle C8-N7-N4-C5 and C8A-N7A-N4A-C5A is 112.13° and −120.04°, respectively). Here, as well, the position of the N7 and N7A atoms is stabilized by the intermolecular hydrogen bonds mentioned above.

### 2.2. In Silico Evaluation of Biological Activity Profiles of Compounds

**Figure 4:**
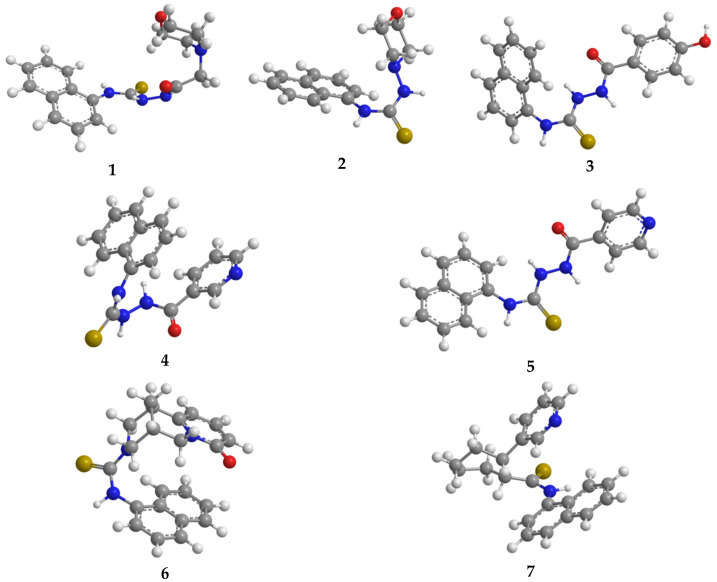
Optimized conformations of compounds **1**–**7** corresponding to configurations with the lowest total energy.

The newly synthesized compounds **1**–**7** were subjected to in silico evaluation of their potential biological activity using AI-driven predictive analytics tools and molecular docking techniques. An in silico evaluation of the newly synthesized compounds (**1**–**7**) was performed using AI-driven predictive analytics and molecular docking to assess their potential biological activity. Initially, the 2D structural formulas of compounds **1**–**7** were converted to IUPAC names (Table S1) and then into 3D models using ChemDraw 22.2.0 and Chem3D 22.2.0 software [19], respectively. Subsequently, conformational analysis was carried out using the online prediction platform Conf-GEM (https://confgem.cmdrg.com, accessed on 6 October 2025), and the most energetically favorable molecular configurations were identified based on MMFF94 calculations [20] (Table S2). The selected conformations were imported into Chem3D and further subjected to energy minimization using the MMFF94 force field (Figure 4).

The obtained 3D molecular models of the synthesized compounds **1**–**7** were subsequently used to predict biological activity based on structural features using the PASS online platform (http://way2drug.com/PassOnline/predict.php, accessed on 8 October 2025), as well as for molecular docking using AutoDock Vina 1.1.2 [21,22].

The PASS online platform (Prediction of Activity Spectra for Substances) utilizes structural descriptors of chemical compounds to predict their potential biological activities, providing Pa and Pi probabilistic values for more than 4000 pharmacological effects, including transporter-related activities, pharmacotherapeutic effects, gene expression regulation, biochemical mechanisms, and others. The predicted types of biological activity for compounds **1**–**7**, as well as their Pa and Pi values, are presented in Table 1.

As can be seen in Table 1, compound **1** demonstrates potential neuropharmacological effects, including treatment of phobic disorders and anticonvulsant activity, alongside moderate antimycobacterial and enzymatic inhibition properties; compound **2** shows a similar neuroactive profile with predicted efficacy against phobic disorders, complemented by antimycobacterial and metabolic regulatory activities; compound **3** exhibits strong antimicrobial and antioxidant potential, with high predicted activity as a taurine dehydrogenase and NADPH oxidase inhibitor; compound **4** is primarily characterized by potent antimycobacterial and antitubercular activity, along with notable inhibition of taurine dehydrogenase and steroid-related enzymes; compound **5** stands out as the most promising candidate, with exceptionally high predicted activity against *Mycobacterium* species and multiple metabolic enzyme targets, particularly taurine dehydrogenase. In general, compounds **1**–**7** exhibit a multifaceted therapeutic profile, with predicted antimycobacterial and antitubercular activities common to all studied compounds. An in silico evaluation of the newly synthesized compounds (**1**–**7**) was performed using AI-driven predictive analytics and molecular docking to assess their potential biological activity. Taurine dehydrogenase inhibition emerges as a dominant feature in compounds **3**–**5**, while neuroactive properties—such as potential treatment of phobic disorders and anticonvulsant activity—are more prominent in compounds **1** and **2**.

As can be seen in Table 1, the results of the PASS online prediction for compounds **1**–**7** demonstrate a notable spectrum of antiviral and hemorheological activities. While compounds **4** and **5** show promising antiviral activity against Picornavirus and Adenovirus (Pa > 0.4), compounds **6** and **7** exhibit significant hemorheological potential through the inhibition of platelet aggregation and vasoprotective effects (Pa up to 0.421).

Since all compounds **1**–**7** exhibited predicted antimycobacterial and antitubercular activities, the next step in the in silico evaluation of their biological potential involved molecular docking using the AutoDock Vina software. Three protein targets were selected for molecular docking simulations, namely: PDB ID **2FUM**, representing the catalytic domain of protein kinase PknB involved in cell growth regulation in Mycobacterium tuberculosis; PDB ID **6HEZ**, corresponding to DprE1, a key flavoprotein in cell wall biosynthesis and a validated target for antimycobacterial agents; and PDB ID **1ENY**, which encodes enoyl-acyl carrier protein reductase (InhA), the primary target of isoniazid in the mycolic acid biosynthesis pathway. For comparative evaluation of antitubercular inhibitory potential, Isoniazid (PubChem CID 3767), Rifampicin (PubChem CID 5360416), and native ligands were selected as reference compounds.

AutoDock Vina employs binding affinity as its scoring function, which estimates the strength and stability of ligand–protein interactions based on predicted free energy of binding; the resulting binding affinities for compounds **1**–**7** docked against the three selected protein targets (PDB IDs: **2FUM**, **6HEZ**, and **1ENY**) are summarized in Table 2.

The docking analysis revealed that several of the tested ligands demonstrated stronger predicted binding affinities toward the selected antitubercular protein targets compared to the reference drugs isoniazid and rifampicin. Docking analysis revealed that several of the tested ligands exhibited stronger predicted binding affinities toward the selected antitubercular protein targets than the reference drugs isoniazid and rifampicin. Notably, compounds **3**–**7** exhibited binding energies consistently lower than −9.0 kcal/mol across at least one of the targets, with compound **6** showing the most pronounced activity (−9.3, −9.0, and −11.3 kcal/mol against **2FUM**, **6HEZ**, and **1ENY**, respectively). These values approach or even surpass the affinities of the native ligands, particularly in the case of **1ENY**, where compound **6** bound more strongly than the native ligand (−11.3 vs. −9.9 kcal/mol). Overall, the results suggest that compounds **3**–**7**, and especially compounds **4** and **6**, possess promising inhibitory potential against multiple protein targets, highlighting their potential as lead candidates for further optimization in antitubercular drug development.

**Figure 5:**
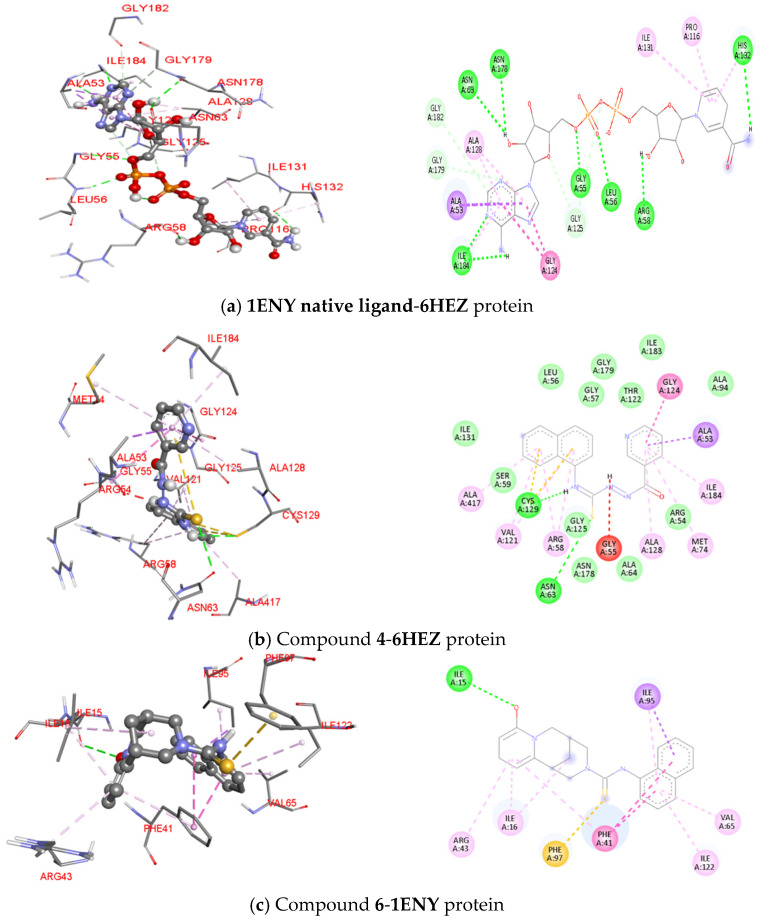
2D and 3D visualization of ligand-amino acid interactions between the **1ENY native ligand**, compounds **4** and **6,** and the selected protein targets (PDB IDs: **2FUM**, **6HEZ**, and **1ENY**), illustrating key binding residues and interaction types within the active sites.

Figure 5 and Table 3 provide a detailed visualization and summary of the molecular interactions between the **1ENY** native ligand, compounds **4** and **6**, and the selected protein targets (PDB IDs: **2FUM**, **6HEZ**, and **1ENY**). These representations highlight the key amino acid residues involved in hydrogen bonding, hydrophobic contacts, and other non-covalent interactions, thereby elucidating the structural basis for the observed binding affinities and supporting the identification of compounds with promising antitubercular potential.

Table 3 presents results of analysis of ligand–protein interactions between 1ENY native ligand, compounds **4** and **6,** and the selected antitubercular targets (PDB IDs: **2FUM**, **6HEZ**, and **1ENY**), revealing diverse and favorable binding profiles. The interaction analysis highlights that compounds **4** and **6** establish diverse and stabilizing contacts with the target proteins, comparable to or exceeding those observed for the 1ENY native ligand. The native ligand engages in multiple conventional hydrogen bonds (e.g., with SER147, TYR94, and ASN67 in **2FUM**; ASN63 and GLY55 in **6HEZ;** and ASP64, ILE95, and GLY96 in **1ENY**), complemented by hydrophobic interactions such as Pi-alkyl contacts with residues like PRO69 and ILE122. Compound **4**, when bound to 6HEZ, forms hydrogen bonds with CYS129 and ASN63, alongside Pi-alkyl and Pi-sulfur interactions, and extensive van der Waals contacts with residues including ILE131, LEU56, and GLY179, suggesting a strong stabilization within the binding pocket. Compound **6** demonstrates particularly favorable interactions: with 2FUM, it establishes hydrogen bonds with ASP36, TYR94, and ASN67, supported by Pi-alkyl contacts with PRO69 and ALA151; with 1ENY, it forms hydrogen bonds with ILE15, Pi-alkyl interactions with ILE122 and ARG43, and additional Pi–Pi stacking with PHE41. These findings indicate that compounds **4** and **6** exploit both polar and nonpolar contacts to achieve high binding affinity, with compound **6** showing a particularly rich interaction profile that may underlie its superior docking scores and potential as a lead antitubercular candidate.

In conclusion, in silico evaluation of compounds **1**–**7** against the selected antitubercular protein targets (PDB IDs: 2FUM, 6HEZ, and 1ENY) demonstrated that several ligands exhibited stronger predicted binding affinities and more diverse interaction profiles than the reference drugs isoniazid and rifampicin. Among them, compounds **3**–**7** showed consistently favorable docking scores, with compound **6** displaying the most potent activity across all targets, surpassing even the native ligand of 1ENY. Detailed interaction analysis revealed that compounds **4** and **6** formed multiple stabilizing hydrogen bonds, hydrophobic contacts, and π-interactions with key active-site residues, supporting their high binding energies. Collectively, these findings suggest that compounds **4** and **6**, represent promising lead candidates for further optimization and development as potential antitubercular agents. Taken together, these results indicate that compounds **4** and **6** are promising leads for further optimization and development as potential antitubercular agents.

### 2.3. Preparation of Inclusion Complexes of New Naphthyl-Containing Thiosemicarbazides with β-Cyclodextrin

**Figure 6:**
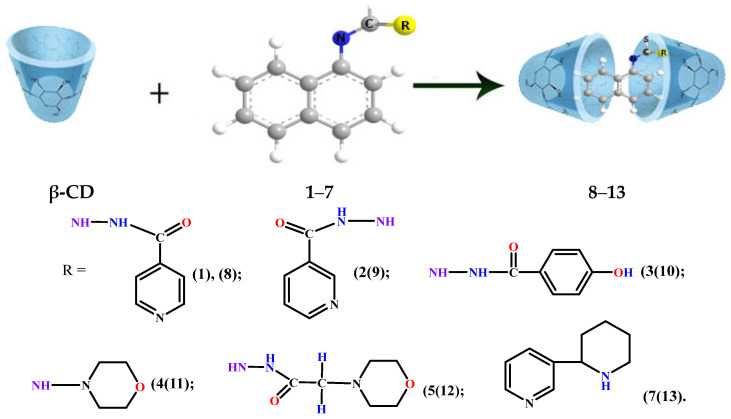
Preparation of inclusion complexes of new naphthyl-containing thiosemicarbazides and thioureas with β-cyclodextrin.

For experimental study of the biological properties of compounds **1**–**7**, they were converted into water-soluble clathrate forms by their encapsulation with β-cyclodextrin in a ratio of (1:2) [23,24,25,26]. The preparation of new complexes of naphthyl-containing thiosemicarbazides with β-cyclodextrin **8**–**14** was carried out in an aqueous alcohol solution. A saturated solution of β-cyclodextrin (CD) in a molar ratio of 1:2 in water at a temperature of 80–90 °C was added drop by drop to a concentrated solution of naphthyl-containing thiosemicarbazides and thioureas **1**–**7** in ethanol (Figure 6). Changes in the structures of the compound in the water-soluble form were studied using NMR spectra (Figures S4–S7 and Table S3).

### 2.4. Thermogravimetric Analysis of Complexes of Inclusions of New Naphthyl-Containing Thiosemicarbazides with β-Cyclodextrin

**Figure 7:**
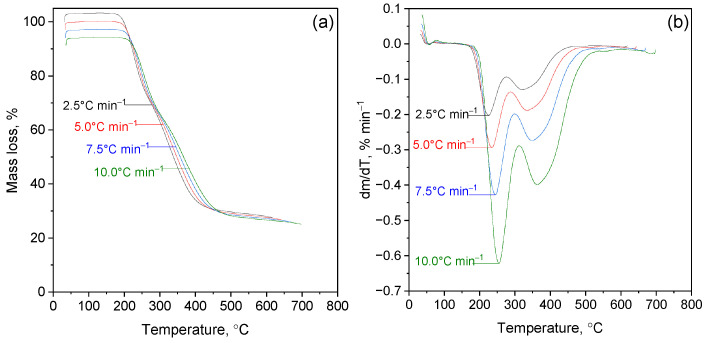
Thermogravimetric (**a**) and differential thermogravimetric (**b**) curves of the β-cyclodextrin inclusion complex with N-(naphthalene-1-yl)-2-nicotinoylhydrazone-1-carbothioamide recorded at heating rates of 2.5, 5.0, 7.5 and 10.0 °C min^−1^ in an inert atmosphere.

The thermal stability of β-CD inclusion complexes with naphthyl-containing thiosemicarbazides and thiourea derivatives was studied by thermogravimetric analysis (TGA) and differential thermogravimetry (DTG) at heating rates of 2.5–10.0 °C min^−1^ in an inert atmosphere (Figure 7; a complete set of data is provided in the Supplementary Materials, Figure S8).

All complexes exhibit a three-stage thermal decomposition profile characteristic typical for supramolecular host–guest systems. The first stage (30–130 °C, 10% mass loss) corresponds to the removal of physically adsorbed and crystallized water, which reflects the destruction of the hydrogen-bound hydrate shell of β-CD. The second stage (200–280 °C) is associated with the thermal degradation of the encapsulated guest molecule. For all complexes, the onset of intense mass loss is shifted to the region of higher temperatures compared with free ligands, which confirms the stabilizing effect of inclusion of β-CD into the hydrophobic cavity. The position and shape of the DTG maxima depend on the ligand structure: derivatives containing hydroxyl and heteroaromatic fragments exhibit narrower peaks indicating enhanced intermolecular interactions (hydrogen bonds and π–π interactions), whereas morpholine-containing compounds are characterized by wider maxima, indicating less stable fixation or partial location of the molecule outside the cavity cyclodextrin. The third stage (280–400 °C) corresponds to the destruction of the macrocyclic β-CD matrix, followed by the breaking of glycosidic bonds and subsequent carbonation. The similarity of thermograms at this stage for all samples indicates the preservation of its own mechanism of thermal decomposition of cyclodextrin, regardless of the nature of the involved ligand.

Thus, thermal analysis confirms that supramolecular encapsulation predominantly affects the kinetics of decomposition of the guest molecule, without changing the fundamental mechanism of thermal degradation of the β-cyclodextrin matrix.

**Figure 8:**
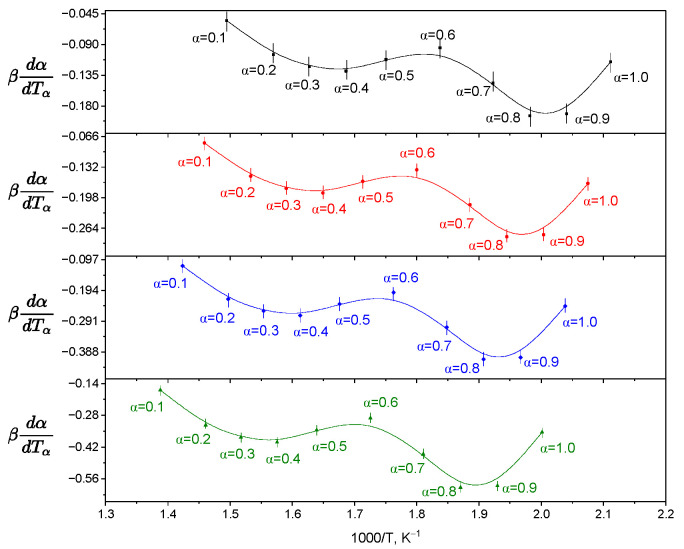
Isoconversion graphs obtained by the Ozawa–Flynn–Wall method for the β-cyclodextrin inclusion complex with N-(naphthalene-1-yl)-2-nicotinoylhydrazono-1-carbothioamide at conversion degrees α = 0.1–1.0 and heating rates 2.5, 5.0 and 10.0 °C min^−1^.

The Ozawa–Flynn–Wall isoconversion method (OFW) [27] was used to determine the effective activation energy without a priori assignment of the kinetic model. The values of *E*_a_ were calculated at various degrees of transformation (α). A representative graph of the dependence is shown in Figure 8, and the corresponding data for all samples are presented in the Supplementary Materials (Figure S9). The obtained values of the activation energy (*E*_a_) demonstrate a systematic dependence on the degree of transformation (α), which excludes the possibility of describing the thermal degradation process within the framework of a single elementary stage with a constant energy barrier (Table 4). In the region of low values of α, reduced values of *E*_a_ are recorded, which corresponds to the course of thermodynamically less costly processes, including desorption of weakly bound water and the initial destruction of the encapsulated guest molecule. As the degree of transformation increases, an increase in the activation energy is observed, reflecting the involvement of more thermostable structural elements in the process, in particular, the destruction of the macrocyclic matrix of β-cyclodextrin and the formation of intermediates with increased stability. As the degree of transformation increases, the activation energy rises, reflecting the involvement of more thermostable structural elements, particularly the degradation of the β-cyclodextrin macrocyclic matrix and the formation of more stable intermediates. Thus, the variation *E*_a_(α) confirms the multi-stage and overlapping character of thermolysis of the studied complexes.

**Figure 9:**
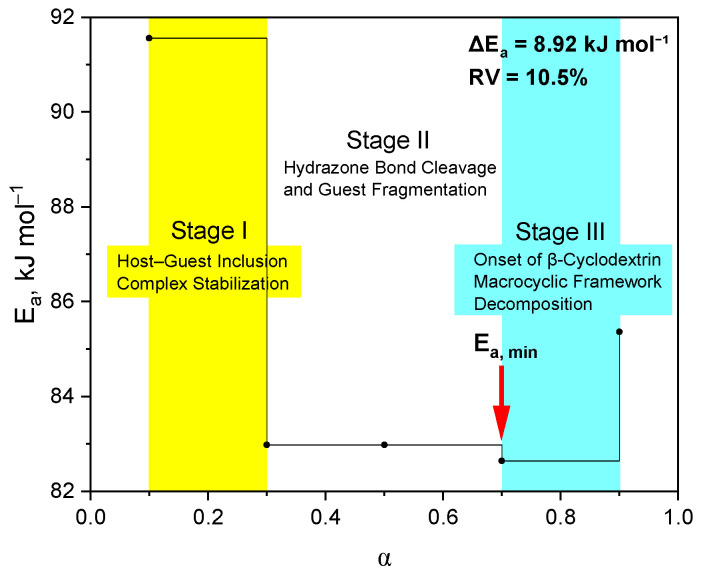
Schematic representation of the multi-stage thermal degradation of the β-cyclodextrin inclusion complex with N-(naphthalene-1-yl)-2-nicotinoylhydrazono-1-carbothioamide.

As can be seen in Figure 9, the dependence *E*_a_(α) confirms the multi-stage character of the thermal destruction of the complex. The amplitude of the change in the activation energy (ΔEa = 8.92 kJ·mol^−1^) and the relative variation (RV = 10.5%) indicate a change in the limiting stage. In the range α = 0.1–0.3, the destruction of guest–host intermolecular interactions is observed, at α = 0.3–0.7, the main destruction of the guest molecule, and at α = 0.7–0.9, the beginning of decomposition of the macrocyclic matrix of β-cyclodextrin. The minimum at α ≈ 0.7 corresponds to the point of the mechanistic transition.

Comparative analysis showed the absence of statistically significant differences between the Friedman [28] and Ozawa–Flynn–Wall methods at most degrees of transformation (*p* > 0.05), which indicates their overall consistency. At the same time, a two-factor analysis of variance revealed a significant influence of the calculation method (*p* < 0.05) and the degree of conversion of α (*p* < 0.001), as well as a statistically significant interaction of factors indicating the dependence of the differences between the methods on the range of α.

**Figure 10:**
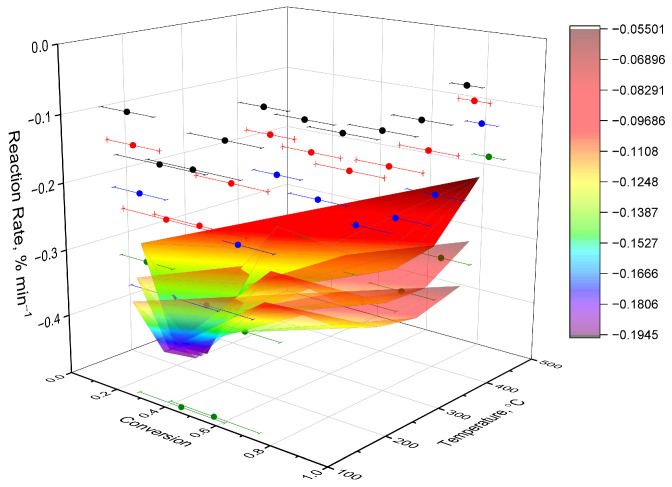
Three-dimensional kinetic surface of the dependence of the rate of thermal decomposition (da/dT) on temperature and degree of transformation (α) for the complex of inclusion of β-CD with N-(naphthalene-1-yl)-2-nicotinoylhydrazono-1-carbothioamide.

To visually characterize the kinetic behavior, three-dimensional surfaces of the reaction rate (da/dT) were constructed in the coordinates “temperature–degree of transformation” (Figure 9; additional surfaces are shown in Figure S10). The obtained surfaces reflect the distribution of reactivity over the entire thermal degradation interval and allow us to identify the contribution of individual stages of the process. To visually characterize the kinetic behavior, three-dimensional surfaces of the reaction rate (da/dT) were constructed in the “temperature–degree of transformation” coordinates (Figure 10; additional surfaces are shown in Figure S10). These surfaces illustrate the distribution of reactivity throughout the entire thermal degradation process and enable the identification of contributions from individual stages.

For all the studied complexes, a dominant maximum was observed on kinetic surfaces, corresponding to the main stage of thermal degradation, as well as less pronounced shoulders reflecting the overlap of successive processes. The position and width of the maxima depend on the ligand structure, which indicates differences in the mechanism of decomposition and the nature of host–guest interactions.

Polar substituents cause more localized and temperature-shifted regions of kinetic activity, indicating increased specific interactions with the beta-cyclodextrin cavity. Derivatives with bulk or conformationally mobile fragments exhibit more extended kinetic surfaces reflecting the structural heterogeneity of the complexes.

Thermogravimetric and kinetic analysis confirm the formation of inclusion complexes and a change in the thermal behavior of compounds when encapsulated with β-CD. The multi-stage process includes removal of bound water, degradation of the guest molecule, and subsequent degradation of the β-CD matrix (see Figures S8–S10).

Thus, thermokinetic parameters (*E_a_*, Δ*E_a_*, *RV*) characterize the strength of guest–host interactions and can be considered as indirect descriptors of the structure–stability–activity relationship.

### 2.5. Antivirus Activity

Naphthyl-containing thiosemicarbazides **1**–**7** have been tested for antiviral activity. As a criterion for the specific antiviral effect of the compounds, the chemotherapeutic index (CTI index) was calculated, determined by the ratio of the average toxic concentration of the substance (TC_50_) to the average effective viral inhibitory concentration (EC_50_).

The virucidal activity [29] of the compounds under study was investigated, which is one of the main approaches to determining the effectiveness of substances with antiviral activity. The dose of the studied substrates was 0.4 mg/chicken embryo. Influenza virus strains were used as model viruses: A/Almaty/8/98 (H3N2); A/Vladivostok/2/09 (H1N1). The acute toxicity of the compounds in a model of 10-day-old chicken embryos was analyzed in the dose range of 0.003–0.4 mg/chicken embryo (0.06–8 mg/kg). It was found that at the maximum dose (0.4 mg/chicken embryo), toxicity (LD_50_) was not manifested; therefore, further study of compounds for antiviral activity was carried out at doses of 0.4 mg/chicken embryo or less. When determining acute toxicity “in vitro”, using a model of 10-day-old chicken embryos, the studied compounds did not show toxic properties at the maximum of the tested doses.

The viral inhibitory activity of compounds for determining the chemical therapeutic index (CTI) was studied at concentrations from 0.0016% to 0.2%, which corresponded to doses of 0.003–0.4 mg per chicken embryo (0.06–8 mg/kg) (Table 5).

As follows from the data in Table 5 of the report, **3** has a high CTI and can be considered as a promising substrate in the search for new anti-influenza drugs.

### 2.6. Hemorheological Activity of Compounds

For the initial assessment of the hemorheological activity of the studied compounds, an in vitro model of high blood viscosity syndrome (HBC) was used, specially designed to reproduce key pathological changes in the rheological properties of blood characteristic of various clinical conditions. The use of this model is due to its high reproducibility, physiological relevance, and the ability to quantify the effects of the tested compounds [30]. The hyperviscosity model was reproduced by incubating blood samples at a temperature of 43.0 °C for 60 min. It was found that exposure to these temperature conditions leads to a significant increase in blood viscosity due to increased aggregation of red blood cells and a decrease in their deformability. Such changes are key mechanisms for the development of hemorheological disorders characteristic of a wide range of vascular and metabolic pathologies [31]. This protocol made it possible to minimize the effect of the solvent on the rheological properties of blood and to objectively evaluate the effect of the tested compounds. The use of this in vitro model made it possible to simulate PCOS in a laboratory experiment, which is an important tool for early detection and pre-screening of substances with potential hemorheological effects (Table S4).

The results of the study demonstrated the effectiveness of the in vitro blood hyperviscosity model for detecting compounds with promising hemorheological activity. Comparison with the control drug (pentoxifylline) demonstrated the expected hemorheological effect, thereby confirming the reliability, reproducibility, and physiological relevance of the chosen screening methodology. Among the seven new compounds studied, 2-(2-morpholinoacetyl)-N-(naphthalene-1-yl)hydrazino-1-carbothioamide **5** and N-2-4(4-hydroxybenzoyl)-(naphthalene-1-yl)hydrazino-1-carbothioamide **3** showed the greatest hemorheological activity, contributing to a decrease in blood viscosity under conditions of induced hyperviscosity in vitro. Statistical processing of the results was carried out using Microsoft Excel software (Microsoft 365), with data representation in the “average ± standard error of the average” format, which meets the requirements of international standards for biomedical data processing.

## 3. Materials and Methods

### 3.1. Reagents and Synthetic Procedures

The following reagents were used: β-Cyclodextrin (99%) (Aldrich, Munich, Germany, mp 270–290 °C with decomp.); isonicotinic acid, nicotinic acid, N-morpholine acetic acid, 4-hydroxybenzoic acid, and aminomorpholine hydrazide were purchased from Sinopharm Chemical Reagent Co., Ltd. (Shanghai, China) “analytically grade”. All solutions were prepared with Elga Millipore deionised water. All reagent solutions were prepared with bidistilled water. The ^1^H and ^13^C NMR spectra were acquired using a JNN-ECA-400 (frequency 399.78 and 100.53 MHz, respectively) instrument (Jeol, Tokyo, Japan) using D_2_O and DMSO-d_6_ as a solvent. Chemical shifts are measured relative to the signals of residual protons or carbon atoms of the deuterated solvent. The FTIR spectrum was measured using a Nicolet iS50 external FTIR spectrometer (Thermo Scientific, Waltham, MA, USA) (KBr, 99%). The elemental analysis (C,H,N) was performed on the EuroVector Elemental Analyser device. The melting temperatures were determined on the SMP10 device. TLC analysis was performed on Silufol UV-254 plates, developed with iodine vapor.

### 3.2. The General Method of Obtaining Naphthyl-Containing Thiosemicarbazides ***1***–***5*** and Thioureas ***6***,***7***

0.0027 mmol of 1-naphthylisothiocyanate in 15 mL of ethanol was added to a mixture of 0.0027 mmol of hydrazide (isonicotinic, nicotinic, N-morpholine acetic, 4-hydroxybenzoic acids), aminomorpholine, cytisine, and anabasine in 15 mL of ethanol while stirring. The reaction mixture was stirred at 90 °C for 3–5 h; the product was filtered, dried, and recrystallized from isopropyl alcohol.

N-(Naphthalene-1-yl)-2-isonicotinoylhydrazino-1-carbothioamide **1**. The yield of **1** was 0.75 g (86.7%), m.p. 221–225 °C.

N-(Naphthalene-1-yl)-2-nicotinoylhydrazino-1-carbothioamide **2**. The yield of **2** was 0.70 g (80.4%), white powder, m.p. 202–204 °C.

2-(2-Morpholinoacetyl)-N-(naphthalene-1-yl)-hydrazino-1-carbothioamide **3**. The yield of **3** was 0.9 g (93%), white powder, m.p. 207–208 °C.

2-(4-hydroxybenzoyl)-N-(naphthalene-1-yl)-hydrazino-1-carbothioamide **4**. The yield of **4** was 0.4 g (44.5%), white powder, m.p. 198–200 °C.

1-Morpholino-3-(naphthalene-1-yl)thiourea **5**. The yield of **5** was 0.62 g (80%), light yellow powder, m.p. 164–165 °C.

1N-(Naphthalene-1-yl)cytisino-1-carbothioamide **6**. The yield of **6** was 0.92 g (94%), white powder, m.p. 260–262 °C.

N-(Naphthalene-1-yl)anabasino-1-carbothioamide **7**. The yield of **7** was 0.65 g (72%), white powder, m.p. 163–165 °C.

### 3.3. The General Procedure for Obtaining Complexes Includes ***8***–***12***

β-cyclodextrin was added to the solution of the corresponding thiosemicarbazide in EtOH in a ratio of (1:2) dissolved in water. The resulting mixture was stirred on a magnetic stirrer at a temperature of 50–70 °C for 4 h. The precipitate was filtered out and placed in a vacuum drying oven at a temperature of 50–55 °C.

The N-(naphthalene-1-yl)-2-isonicotinoylhydrazino-1-carbothioamide inclusion complex with β-cyclodextrin **8** was obtained from 0.0003 M of N-(naphthalene-1-yl)-2-isonicotinoylhydrazino-1-carbothioamide **1** in 20 mL of dimethyl sulfoxide and 0.0003 M of β-cyclodextrin in 10 mL of water. The yield was 0.34 g (79%), m.p. 221–240 °C.

The N-(naphthalene-1-yl)-2-nicotinoylhydrazino-1-carbothioamide inclusion complex with β-cyclodextrin **9** was obtained similarly to compound **8** from 0.00015 M of N-(naphthalene-1-yl)-2-nicotinoylhydrazino-1-carbothioamide **2** in 10 mL of ethanol and 0.0003 M β-cyclodextrin in 15 mL of water. The yield was 0.17 g (77.9%), m.p. 220–238 °C.

The inclusion complex of 2-(4-hydroxybenzoyl)-N-(naphthalene-1-yl)-hydrazino-1-carbothioamide with β-cyclodextrin **10** was obtained similarly to compound **8** from 0.000148 M 2-(4-hydroxybenzoyl)-N-(naphthalene-1-yl)-hydrazino-1-carbothioamide **4** in 10 mL of ethanol and 0.000148 M of β-cyclodextrin in 10 mL of water. The yield was 0.20 g (94%), m.p. 225–250 °C.

The inclusion complex of 2-(2-morpholinoacetyl)-N-(naphthalene-1-yl)-hydrazino-1-carbothioamide with β-cyclodextrin **11** was obtained similarly to compound **8** from 0.0006 M 2-(2-morpholinoacetyl)-N-(naphthalene-1-yl)-hydrazino-1-carbothioamide **3** in 50 mL of ethanol and 0.0006 M of β-cyclodextrin in 10 mL of water. The yield was 0.73 g (83.2%), m.p. 225–250 °C.

The 1-morpholino-3-(naphthalene-1-yl)thiourea inclusion complex with β-cyclodextrin **12** was obtained similarly to compound **8** from 0.00034 M 1-morpholino-3-(naphthalene-1-yl)thiourea **5** in 20 mL of ethanol and 0.00034 M β-cyclodextrin in 10 mL of water. The yield was 0.72 g (75.4%), m.p. 248–257 °C.

The N-(Naphthalene-1-yl)anabasino-1-carbothioamide inclusion complex with β-cyclodextrin **13** was obtained similarly to compound **8** from 0.00034 M N-(Naphthalene-1-yl)anabasino-1-carbothioamide **7** in 20 mL of ethanol and 0.00034 M β-cyclodextrin in 10 mL of water. The yield was 0.72 g (93.91%), m.p. 298–316 °C.

### 3.4. In Silico Evaluation of Biological Activity Profiles of Compounds

In silico evaluation of the biological activity potential was performed using AI-driven predictive analytics tools and molecular docking techniques. Initially, the ChemOffice package (PerkinElmer, Shelton, CT, USA) [19] was used to convert the 2D structural formulas of compounds **1**–**5** into IUPAC names (ChemDraw) and 3D models (Chem3D) in *.smi* format. Subsequently, online prediction platform Conf-GEM (https://confgem.cmdrg.com, accessed on 6 October 2025) was used for conformational analysis, and the most energetically favorable molecular configurations were identified based on MMFF94 calculations [20]. The selected conformations in .mol format were imported into Chem3D and further subjected to energy minimization using the MMFF94 force field. The obtained molecular geometries were saved in .mol format for PASS analysis and also converted to the .pdb format using Chem3D for subsequent molecular docking.

PASS (Prediction of Activity Spectra for Substances) analysis [21] was performed using PASS online platform, available at http://way2drug.com/PassOnline/predict.php, accessed on 8 October 2025. Predictive outputs from PASS are expressed as Pa and Pi probability scores [22,32]. The Pa score, or probability “to be active,” ranges from 0 to 1 and estimates the likelihood that a compound belongs to the class of biologically active substances, based on structural similarity to known actives in the PASS training set. Conversely, the Pi score, or probability “to be inactive” also ranging from 0 to 1, reflects the likelihood that the compound resembles structures typical of inactive substances. In drug discovery and virtual screening, researchers often apply a threshold—commonly 0.5 or higher—considering compounds with Pa values equal to or above this threshold as potentially active. However, this threshold is flexible and may be adjusted depending on the specific aims of the study, the acceptable level of risk, and the strategic priorities of the research team. Therefore, for the purposes of our study, we selected the top six predicted activities based on descending Pa values (probability “to be active”).

Molecular docking simulations was performed using AutoDock Vina 1.1.2 and AutoDock MGL Tools 1.5.7 software (Molecular Graphics Laboratory, the Scripps Research Institute, La Jolla, CA, USA) [33,34]. Three validated antitubercular protein targets were retrieved from the Protein Data Bank (PDB, https://www.rcsb.org, accessed on 1 March 2026) for molecular docking studies: PDB ID 2FUM corresponds to the catalytic domain of Mycobacterium tuberculosis serine/threonine-protein kinase PknB, a key regulator of bacterial cell growth, division, and signal transduction; PDB ID 6HEZ represents decaprenylphosphoryl-β-D-ribose 2′-epimerase (DprE1), a flavoprotein essential for arabinan biosynthesis in the mycobacterial cell wall and a well-established target for tuberculosis therapy; and PDB ID 1ENY encodes enoyl-acyl carrier protein reductase (InhA), a crucial enzyme in the fatty acid elongation cycle of mycolic acid biosynthesis and the primary target of the first-line antitubercular drug isoniazid. To assess the antitubercular inhibitory potential of the synthesized compounds, Isoniazid (PubChem CID: 3767) and Rifampicin (PubChem CID: 5360416) were chosen as benchmark reference drugs.

The preparation of the molecular structures of the proteins included the steps of removing native ligands and water molecules, protonation, and creating a binding site. The preparation of the ligands included MMFF94 energy minimization using the Chem3D software. The molecular preparation of proteins involved the removal of native ligands and water molecules, protonation, and the establishment of the binding site. Ligands were prepared by performing MMFF94 energy minimization using Chem3D software. The position of the binding site was determined based on PDB data, and the following grid coordinates of the receptor active site were used: (x = 58.3411, y = −1.3335, z = −40.1378) for the **2FUM**, (x = 8.4959, y = −12.9209, z = 36.9891) for the structure of **6HEZ** and (x = −2.7216, y = 33.8826, z = 14.1626) for the structure of **1ENY** target proteins. Based on the docking results, a comparative analysis of the binding affinities and intermolecular interactions between the studied compounds and the proteins was performed. The study of non-covalent interactions between proteins and ligands was performed using the BIOVIA Discovery Studio Visualizer 2021 program.

### 3.5. Statistical Analysis

All determinations were conducted in triplicate, and all results were calculated as mean ± standard deviation (SD). Pearson linear correlation coefficients were used to assess relationships among variables, antioxidant capacity results and total phenolic content, and were performed with SPSS software (version 19.0; SPSS Inc., Chicago, IL, USA). Using this software, one-way ANOVA, followed by Tukey’s HSD test, was performed to analyze the significant differences between data (*p* < 0.05).

### 3.6. Study of Antivirus Activity

Virucidal activity of compounds **1**–**7** was tested at a dose of 0.4 mg/chicken embryo. Influenza virus strains were used as model viruses: A/Almaty/8/98 (H3N2); A/Vladivostok/2/09 (H1N1). The acute toxicity of the compounds in a model of 10-day-old chicken embryos was analyzed in the dose range of 0.003–0.4 mg/chicken embryo (0.06–8 mg/kg).

The direct virucidal activity of the compounds was evaluated by incubating viral suspensions with equal volumes of the tested substances for 30 min at 37 °C. Following incubation, the mixtures were subjected to tenfold serial dilutions in sterile phosphate-buffered saline (PBS) to attenuate compound activity through dilution. The resulting dilutions were subsequently inoculated into the allantoic cavities of embryonated eggs or applied to monolayers of susceptible cell cultures.

After an incubation period ranging from 24 to 72 h, depending on the specific viral strain, infectious titers were determined and expressed as EID_50_/mL or TCID_50_/mL, calculated using the Reed–Muench method. Control samples were treated with sterile PBS (pH 7.2) under identical experimental conditions. Virucidal efficacy was quantified as the reduction in viral titer, expressed as the log_10_ difference between treated and untreated samples. All experiments were performed in triplicate across three independent experimental series.

Pharmacokinetic properties, metabolite profiles, and drug-likeness of the investigated compounds were predicted using in silico modeling approaches. Pharmacokinetic parameters and medicinal chemistry compatibility were assessed using the SwissADME web server and the ADMETlab 2.0 platform. Drug-likeness was evaluated according to established criteria, including Lipinski’s rule of five, as well as Weber’s and Ghose’s rules, which consider parameters such as the number of rotatable bonds and polar surface area.

### 3.7. Study of Hemorheological Activity

The blood viscosity of male rats was measured using a Brookfield DV2T rotary viscometer, which made it possible to record changes in viscosity at different spindle speeds (2, 4, 6, 8, 12, 20, and 40 vol·s^−1^). This approach provides a comprehensive assessment of the effect of compounds on both the deformability of erythrocytes at high shear rates and their aggregation properties at low rates [31]. Blood was taken from healthy male Wistar rats, which ensured the standardization of biological material. After determining the initial viscosity values, the samples were incubated with the test substances (final concentration 10^−1^ g/mL) in DMSO solution at 43.00 °C for 60 min. The control samples contained an equivalent amount of DMSO without active compounds.

## 4. Conclusions

The interaction of naphthyl-containing thiosemicarbazides with β-cyclodextrin resulted in the formation of their inclusion complexes. The conducted thermoanalytical study demonstrated that the structure of the included ligand, as well as the type of cyclodextrin, significantly affects the thermal stability and kinetic parameters of the decomposition of the inclusion complex. The viral inhibitory activity of the compounds was studied to determine their chemical therapeutic index. New water-soluble naphthyl-containing thiosemicarbazides appear to be promising candidates for further study of their antibacterial properties due to their consistently strong interaction with key anti-tuberculosis targets. All new compounds exhibited the ability to modulate both red blood cell deformability and aggregation, highlighting their potential as candidates for further investigation. The obtained compounds represent a promising scaffold for the development of antiviral agents that exhibit potency at micro- and nanomolar concentrations.

## Figures and Tables

**Table 1:** PASS online predicted types of biological activity for compounds **1**–**7**.

Compound		Types of Biological Activity	Pa *	Pi **
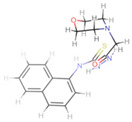	**1**	Phobic disorders treatment	0.728	0.063
		Anticonvulsant	0.634	0.015
		PfA-M1 aminopeptidase inhibitor	0.600	0.005
		Antimycobacterial	0.578	0.011
		HMGCS2 expression enhancer	0.554	0.022
		Glyoxylate reductase inhibitor	0.526	0.023
		Antiviral (Adenovirus)	0.234	0.159
		Antiviral (Poxvirus)	0.195	0.167
		Vasoprotector	0.256	0.233
		Antihemorrhagic	0.125	0.090
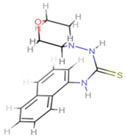	**2**	Phobic disorders treatment	0.702	0.075
		Antituberculosic	0.620	0.005
		HMGCS2 expression enhancer	0.627	0.015
		Antimycobacterial	0.582	0.011
		Glyoxylate reductase inhibitor	0.511	0.026
		Heat shock protein 27 antagonist	0.466	0.026
		Antiviral (Adenovirus)	0.253	0.135
		Antiviral (Influenza)	0.214	0.176
		Antiviral (Picornavirus)	0.276	0.269
		Antiviral (Poxvirus)	0.231	0.116
		Antihemorrhagic	0.115	0.115
		Vasoprotector	0.256	0.233
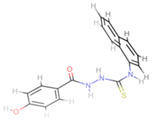	**3**	Taurine dehydrogenase inhibitor	0.804	0.012
		Antimycobacterial	0.787	0.004
		NADPH oxidase inhibitor	0.785	0.002
		Antituberculosic	0.767	0.003
		PfA-M1 aminopeptidase inhibitor	0.719	0.003
		Corticosteroid side-chain-isomerase inhibitor	0.698	0.009
		Antiviral (Adenovirus)	0.328	0.068
		Antiviral (Influenza)	0.319	0.078
		Antiviral (Picornavirus)	0.329	0.184
		Antiviral (Poxvirus	0.341	0.042
		Antihemorrhagic	0.135	0.072
		Vasoprotector	0.253	0.239
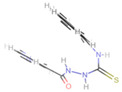	**4**	Antimycobacterial	0.787	0.004
		Antituberculosic	0.762	0.004
		Taurine dehydrogenase inhibitor	0.754	0.021
		PfA-M1 aminopeptidase inhibitor	0.684	0.004
		Corticosteroid side-chain-isomerase inhibitor	0.677	0.010
		Ethanolamine oxidase inhibitor	0.572	0.004
		Antiviral (Adenovirus)	0.269	0.118
		Antiviral (Picornavirus)	0.292	0.240
		Antiviral (Poxvirus)	0.286	0.067
		Antithrombocytopenic	0.191	0.02
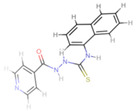	**5**	Taurine dehydrogenase inhibitor	0.875	0.005
		Antimycobacterial	0.836	0.003
		Antituberculosic	0.813	0.003
		Amine dehydrogenase inhibitor	0.751	0.005
		Glutamine-phenylpyruvate transaminase inhibitor	0.742	0.008
		Isopenicillin-N epimerase inhibitor	0.736	0.004
		Antiviral (Adenovirus)	0.309	0.082
		Antiviral (Influenza)	0.216	0.174
		Antiviral (Picornavirus)	0.417	0.097
		Antiviral (Poxvirus)	0.417	0.027
		Antithrombocytopenic	0.182	0.002
		Hemostatic	0.170	0.148
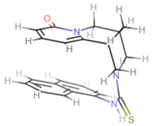	**6**	Nicotinic alpha2beta2 receptor antagonist	0.903	0.003
		Polarization stimulant	0.820	0.002
		Nicotinic alpha4beta2 receptor antagonist	0.813	0.002
		Membrane permeability inhibitor	0.630	0.072
		Nicotinic alpha6 receptor agonist	0.470	0.004
		Platelet aggregation inhibitor	0.421	0.017
		Antiviral (Influenza A)	0.318	0.031
		Platelet aggregation inhibitor	0.421	0.017
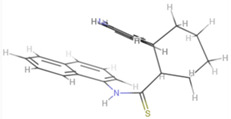	**7**	Nicotinic alpha6beta3beta4alpha5 receptor antagonist	0.819	0.008
		Nicotinic alpha2beta2 receptor antagonist	0.804	0.009
		Mucomembranous protector	0.722	0.047
		Nicotinic alpha4beta4 receptor agonist	0.670	0.024
		Spasmolytic, urinary	0.648	0.017
		Antituberculosic	0.314	0.063
		Antiviral (HIV)	0.135	0.085
		Vasoprotector	0.350	0.108

* Pa—is the probability of being active, ** Pi—is the probability of being inactive.

**Table 2:** Predicted binding affinities (kcal/mol) of compounds **1**–**7** with selected antitubercular protein targets (PDB identifiers: **2FUM**, **6HEZ** and **1ENY**).

Compound	Binding Affinities (kcal/mol)		
	2FUM	6HEZ	1ENY
Reference compounds			
Isoniazid	−5.2	−6.2	−6.0
Rifampicin	−8.4	−7.1	−8.1
2FUM native ligand	−6.6	−7.9	−7.6
6HEZ native ligand	−8.7	−9.5	−9.7
1ENY native ligand	−9.0	−11.9	−9.9
Ligands			
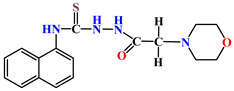 **1**	−7.8	−8.6	−9.8
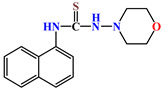 **2**	−7.3	−8.8	−8.9
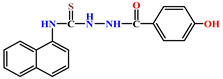 **3**	−8.4	−9.3	−10.0
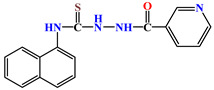 **4**	−8.1	−9.7	−9.3
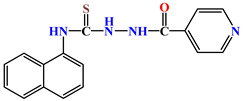 **5**	−8.2	−9.1	−10.1
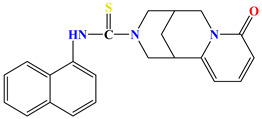 **6**	−9.3	−9.0	−11.3
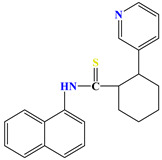 **7**	−7.6	−8.4	−10.6

**Table 3:** Key ligand–protein interactions between the **1ENY** native ligand, compounds **4** and **6,** and target proteins (PDB IDs: **2FUM**, **6HEZ**, and **1ENY**).

	Complex	1ENY Native Ligand-Receptor			Ligand-Receptor		
Interactions		2FUM	6HEZ	1ENY	4-6HEZ	6-2FUM	6-1ENY
Conventional Hydrogen Bond		SER147,TYR94,TYR75,THR149,GLU93,ASN67	ILE184, ASN63, ASN178, GLY55, LEU56, LEU56, ARG58	VAL65, ASP64, ILE95, ILE21, LYS165, ILE194, GLY96	CYS129, ASN63	ASP36,TYR94,ASN67	ILE15
Pi-Alkyl		ALA151,PRO69,PRO69	ALA128, ILE131, PRO116	PHE41, ILE122, ALA191	ALA417, VAL121, ARG58, ALA128, ILE184, MET74	PRO69, ALA151	ILE122,VAL65,ILE21, ARG43
Pi-Sigma		-	ALA53	-	ALA53	-	ILE95
Pi-Sulfur		-	-	-	CYS129	-	PHE97
Carbon Hydrogen Bond		LEU66	GLY179, GLY182, GLY125	GLY96	-	TYR94	ILE15
Unfavorable Donor-Donor		GLU93, THR149		GLN66, TYR196	GLY55	-	-
Vander Waals		-	-	-	ILE131, LEU56, GLY57, GLY179, ILE183, THR122, ALA94, ARG54, ALA64, ASN128, SER59	-	-
Pi-Pi stacked		-	-	-	-	-	PHE41

**Table 4:** Activation energy (*E_a_*, kJ mol^−1^) as a function of the degree of transformation (α), calculated by the Friedman and Ozawa–Flynn–Wall methods for the studied compounds.

Sample/*α*	Method	0.1	0.3	0.5	0.7	0.9
β-CD with 2-(morpholinoacetyl)-N-(naphtha-lene-1-yl)girdrazine-1-carbothioamide	Friedman	87.15 ± 1.20	85.14 ± 1.20	83.87 ± 1.12	82.40 ± 0.90	83.19 ± 1.32
	OFW	90.06 ± 1.00	87.57 ± 1.41	86.20 ± 1.13	81.3 ± 1.71	84.22 ± 1.21
β-CD with 1-morpholino-3-(naphthalene-1-yl) thiourea	Friedman	86.36 ± 1.30	84.42 ± 1.23	83.90 ± 1.02	83.65 ± 1.54	84.46 ± 1.70
	OFW	88.61 ± 1.41	86.12 ± 1.31	84.58 ± 1.41	83.48 ± 1.90	84.01 ± 1.50
β-CD with 2-(4-hydroxybenzoyl)-N-(naphthalene-1-yl)-hydrazino-1-carbothioamide	Friedman	91.56 ± 1.10	82.98 ± 1.00	83.28 ± 1.20	82.64 ± 1.10	85.36 ± 1.00
	OFW	94.13 ± 1.51	84.75 ± 1.30	85.51 ± 1.20	85.18 ± 1.70	89.57 ± 2.00
β-CD with N-(naphthalene-1-yl)-anabasino-1-carbothioamide	Friedman	86.37 ± 1.00	84.92 ± 1.10	84.59 ± 0.90	84.16 ± 1.00	83.43 ± 1.10
	OFW	88.98 ± 1.10	86.01 ± 1.21	86.68 ± 1.11	86.27 ± 1.30	85.03 ± 1.21

Note: All values are given as the mean ± standard deviation (*n* = 3).

**Table 5:** Viral inhibitory activity of compounds in relation to influenza viruses.

Compound	Chemical Therapeutic Index of the Drug	
	A/Almaty/8/98 (H3N2)	A/Vladivostok/2/09 (H1N1)
**1**	12	15
**2**	2	5
**3**	25	40
**4**	9	7
**5**	5	9
Tamiflu	10.3	11
Remantadine	29.9	30

## Data Availability

The data that support the findings of this study are available within the article and the Supplementary Materials. Further data are available from the corresponding author upon reasonable request.

## Supplementary Materials

The following supporting information can be downloaded at https://www.mdpi.com/article/10.3390/molecules31081290/s1, Figure S1: IR spectras of N-(naphthalene-1-yl)-2-nicothionyl-hydrazino-1-carbothioamide **2** (a) and N-2-(2-morpholinoacetyl)-(naphthalene-1-yl)-hydrazino-1-carbothioamide **5** (b); Figure S2: NMR ^1^H (a), ^13^C (b), COSY (^1^H–^1^H) (c), HMQC (^1^H–^13^C) (d) spectra’s of 1-morpholine-3-(naphthalen-1-yl)thiourea **4**; Figure S3: NMR ^1^H (a), ^13^C (b), COSY (^1^H–^1^H) (c), HMQC (^1^H–^13^C) (d) and HMBC (e) spectras of N-(naphthalene-1-yl)anabasino-1-carbothioamide **7**; Table S1: Compounds **1**–**7** and their IUPAC names; Table S2: Conformers of **1**–**7** compounds and their MMFF94 steric energies; Figure S4: ^1^H NMR spectrum of β-CD (DMSO); Figure S5: COSY (^1^H–^1^H) NMR spectrum of β-CD (DMSO); Figure S6: HSQC (^1^H–^13^C) NMR spectrum of β-CD (DMSO); Figure S7: HMBC (^1^H–^13^C) NMR spectrum of β-CD (DMSO); Table S3: Chemical shifts of ^1^H and ^13^C cyclodextrin nuclei in the free state (δ0) and as part of the inclusion complex with (δ); Figure S8: Thermogravimetric (TGA) and differential thermogravimetric (DTG) curves of inclusion complexes with β-cyclodextrins, recorded at heating rates of 2.5, 5.0, 7.5, and 10.0 °C/min: (a) Inclusion complex of 2-(morpholinoacetyl)-N-(naphthalen-1-yl)hydrazine-1-carbothioamide with β-CD; (b) Inclusion complex of 1-morpholino-3-(naphthalen-1-yl)thiourea with β-CD; (c) Inclusion complex of 2-(4-hydroxybenzoyl)-N-(naphthalen-1-yl)-hydrazino-1-carbothioamide with β-CD; (d) Inclusion complex of N-(naphthalen-1-yl)-anabasine-1-carbothioamide with β-CD; (e) Inclusion complex of N-(naphthalene-1-yl)-2-nicotinoylhydrazono-1-carbothioamide with β-CD; (f) Inclusion complex of N-(naphthalene-1-yl) 2-isonicotinoylhydrazono-1-carbothioamide with β-CD; Figure S9: Dependences of logβ on 1/T, constructed according to the Ozawa–Flynn–Wall method for calculating the activation energy of thermal decomposition of inclusion complexes with β-cyclodextrins at different degrees of transformation (α = 0.1–0.9). The calculations were performed based on TGA data obtained at heating rates of 2.5, 5.0, 7.5, and 10.0 °C/min: (a) Inclusion complex of 2-(morpholinoacetyl)-N-(naphthalen-1-yl)hydrazine-1-carbothioamide with β-CD; (b) Inclusion complex of 1-morpholino-3-(naphthalen-1-yl)thiourea with β-CD; (c) Inclusion complex of 2-(4-hydroxyphenyl)-N-(naphthalen-1-yl)-hydrazino-1-carbothioamide with β-CD; (d) Inclusion complex of N-(naphthalen-1-yl)-anabasine-1-carbothioamide with β-CD; (e) Inclusion complex of N-(naphthalene-1-yl)-2-nicotinoylhydrazono-1-carbothioamide with β-CD; (f) Inclusion complex of N-(naphthalene-1-yl) 2-isonicotinoylhydrazono-1-carbothioamide with β-CD; Figure S10: Three-dimensional dependences of dα/dT on temperature T and degree of conversion α for inclusion complexes with β-cyclodextrins, calculated at various rates of 5.0; 7.5; 10.0, and 12.5 °C min^−1^: (a) Inclusion complex of 2-(morpholinoacetyl)-N-(naphthalen-1-yl)-hydrazine-1-carbothioamide with β-CD; (b) Inclusion complex of 1-morpholino-3-(naphthalen-1-yl)thiourea with β-CD; (c) Inclusion complex of 2-(4-hydroxyphenyl)-N-(naphthalen-1-yl)-hydrazino-1-carbothioamide with β-CD; (d) Inclusion complex of N-(naphthalen-1-yl)-anabasine-1-carbothioamide with β-CD; (e) Inclusion complex of N-(naphthalene-1-yl)-2-nicotinoylhydrazono-1-carbothioamide with β-CD; (f) Inclusion complex of N-(naphthalene-1-yl)2-isonicotinoylhydrazono-1-carbothioamide with β-CD; Table S4: Effect of samples **1**–**7** on blood viscosity (mPa*s) at different spindle rotation speeds on an in vitro blood hyperviscosity model (incubation at 43 °C).
