# Supplementary material for: Water-Soluble Inclusion Complexation of Naphthyl-Containing Thiosemicarbazides and Thioureas with β-Cyclodextrin
Source: Molecules. 2026 Apr 15;31(8):1290. doi: 10.3390/molecules31081290 (PMC13119154; doi:10.3390/molecules31081290)
Supplement: Supplementary file 1 [file molecules-31-01290-s001.zip › molecules-4206708-supplementary.pdf]

Water-Soluble Inclusion Complexation of Naphthyl-Containing Thiosemicarbazides and Thioureas with β-Cyclodextrin

Oralgazy A. Nurkenov[1,2], Zainulla M. Muldakhmetov[1], Serik D. Fazylov[1,*], Anel Zh. Mendibayeva[1,*], Irina A. Pustolaikina[3], Akmaral Zh. Sarsenbekova[3], Olzhas T. Seilkhanov[4], Ardak K. Syzdykov[1,2], Saule K. Kabiyeva[2], Zhangeldy S. Nurmaganbetov[1]

[1] Laboratory of Synthesis of Biologically Active Substances, Institute of Organic Synthesis and Coal Chemistry of the Republic of Kazakhstan, Karaganda 100008, Kazakhstan; nurkenov_oral@mail.ru (O.A.N.); iosu.rk@mail.ru (Z.M.M.); faz8990@mail.ru (S.D.F.); anenyawa@mail.ru (A.Zh.M.); ardak.syzdykov.96@inbox.ru (A.K.S.); nzhangeldy@yandex.ru (Zh.S.N.)
[2] Department of Chemical Technology and Ecology, Karaganda Industrial University, Temirtau 101400, Kazakhstan; ardak.syzdykov.96@inbox.ru (A.K.S.); kabieva.s@mail.ru (S.K.K.)
[3] Department of Physical and Analytical Chemistry, Karaganda National Research University named after Academician E.A. Buketov, Karaganda, 100024, Kazakhstan; Pustolaikina.I@karnu-buketov.edu.kz (I.A.P.); chem_akmaral@mail.ru (A.Zh.S.)
[4] Laboratory of Engineering Profile NMR-Spectroscopy, Sh. Ualikhanov Kokshetau University, Kokshetau 120000, Kazakhstan; seilkhanov@mail.ru (O.T.S.)
* Correspondence: faz8990@mail.ru (S.D.F.); anenyawa@mail.ru (A.Zh.M.)

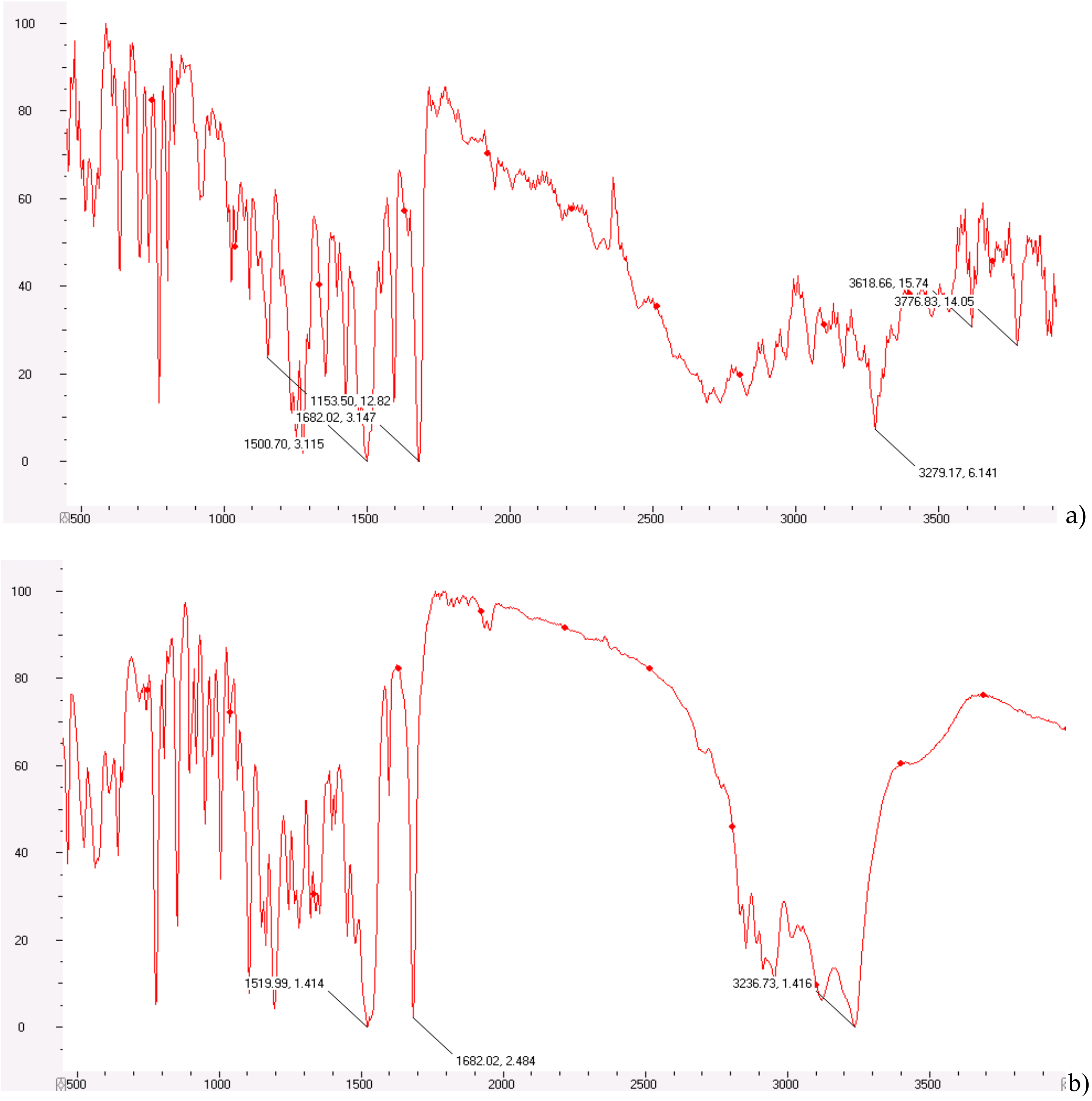

Figure S1. IR spectras of N-(naphthalene-1-yl)-2-nicothionyl- hydrazino-1-carbothioamide **2** (a) and N-2-(2-morpholinoacetyl) -(naphthalene-1-yl)-hydrazino-1-carbotioamide **5** (b)

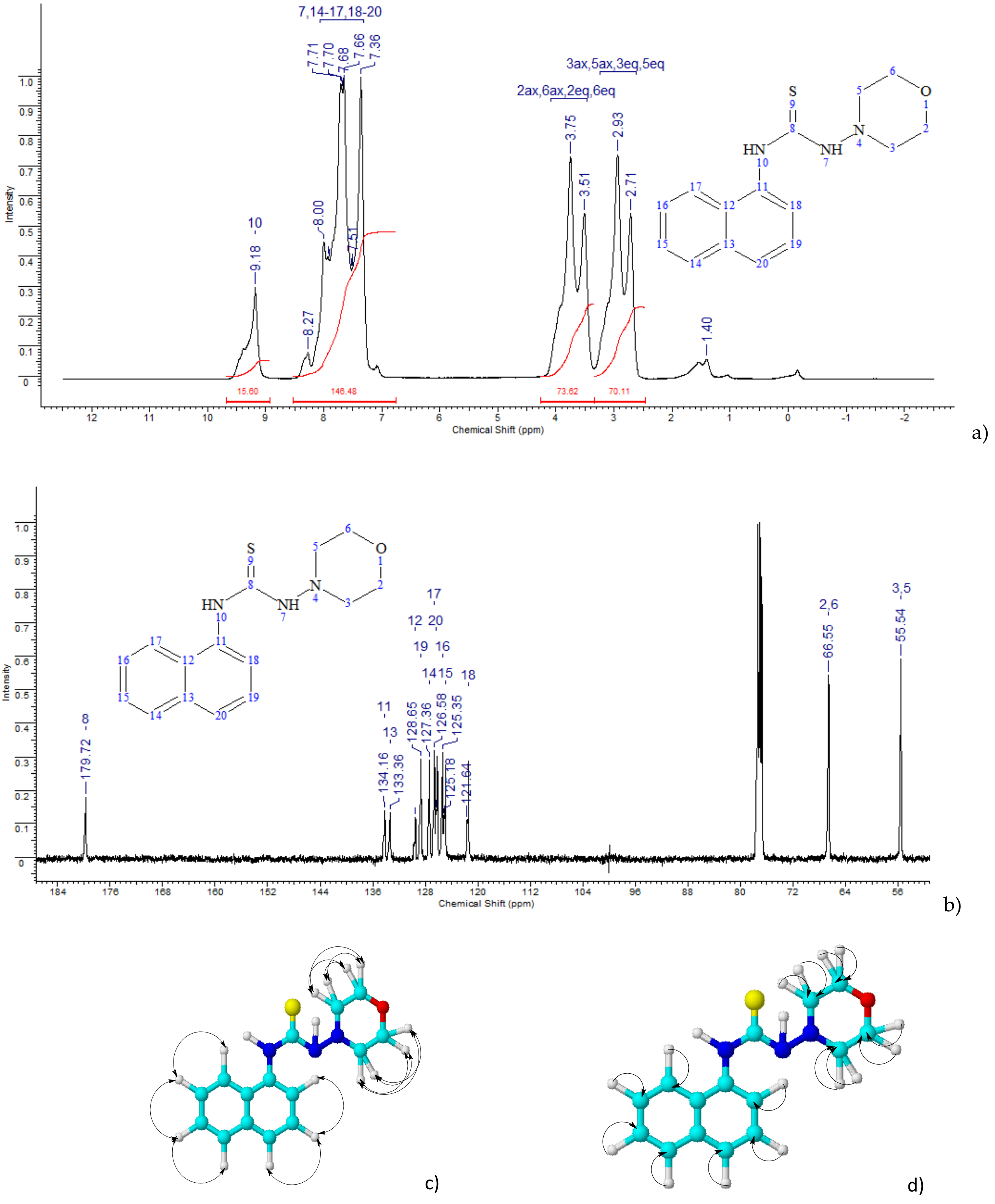

Figure S2. NMR ^{1}H (a), ^{13}C (b), COSY (^{1}H-^{1}H) (c), HMQC (^{1}H-^{13}C) (d) spectras of 1-morpholine-3-(naphthalen-1-yl)thiourea **4**

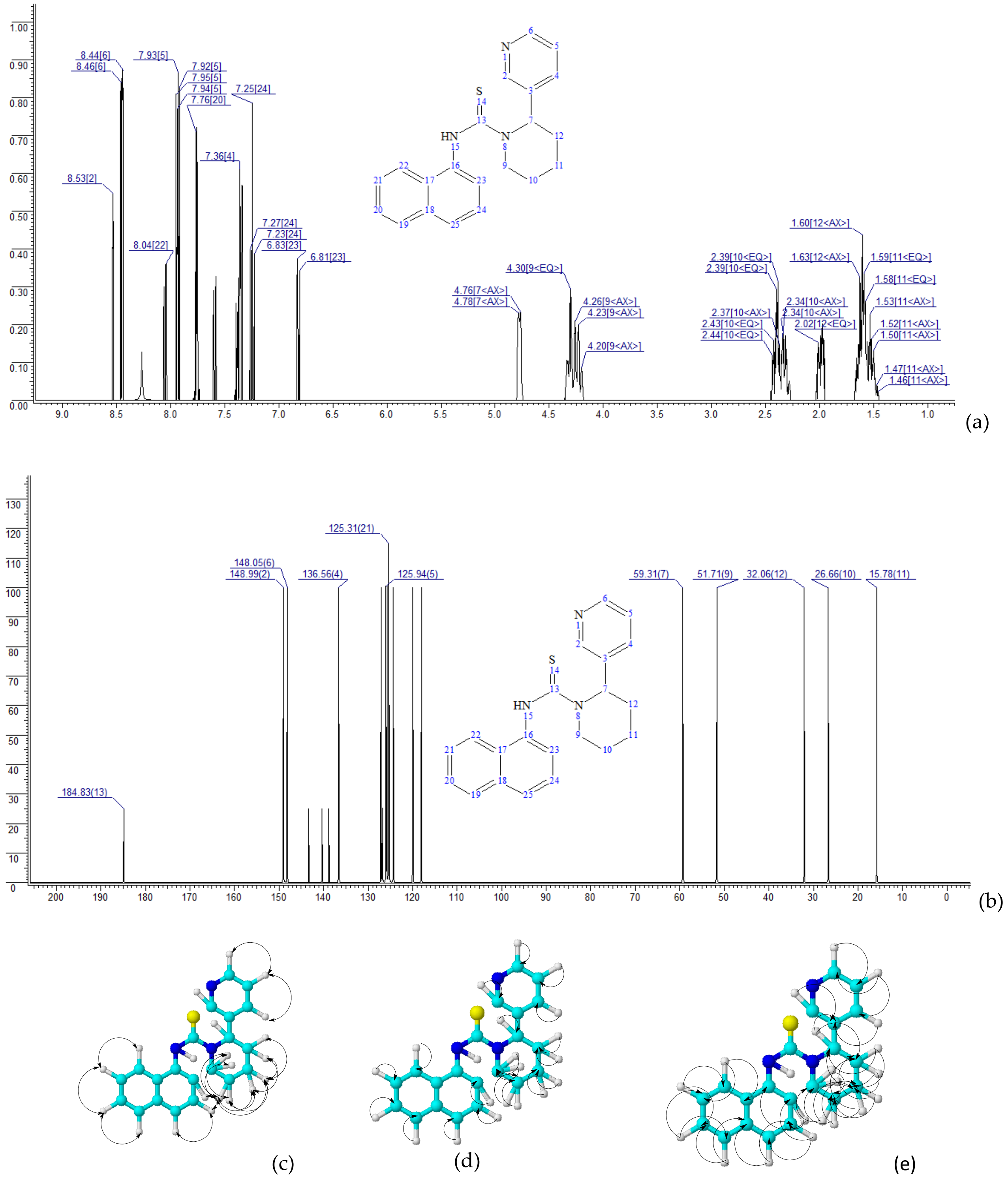

Figure S3. NMR ¹H (a), ¹³C (b), COSY (¹H-¹H) (c), HMQC (¹H-¹³C) (d) and HMBC (e) spectras of N-(naphthalene-1-yl)anabasino-1-carbotioamide **7**

Table S1. Compounds **1-7** and their IUPAC names

№	Compound	IUPAC names
1	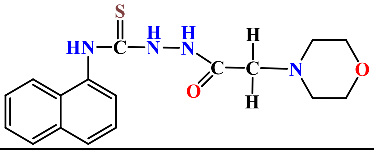	2-(2-morpholinoacetyl)-N-(naphthalen-1-yl)hydrazine-1-carbothioamide
2	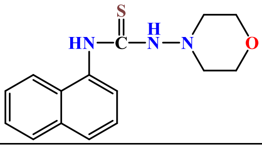	1-morpholino-3-(naphthalen-1-yl)thiourea
3	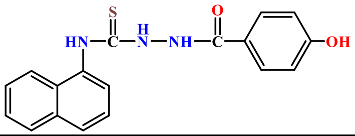	2-(4-hydroxybenzoyl)-N-(naphthalen-1-yl)hydrazine-1-carbothioamide
4	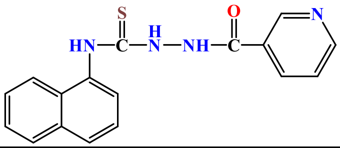	N-(naphthalen-1-yl)-2-nicotinoylhydrazine-1-carbothioamide
5	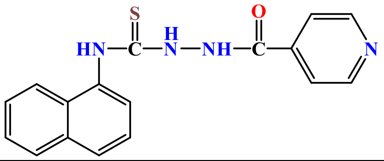	2-isonicotinoyl-N-(naphthalen-1-yl)hydrazine-1-carbothioamide
6	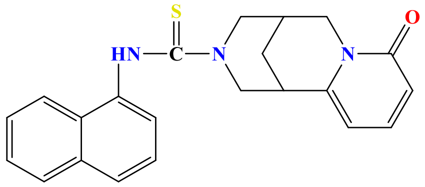	N-(naphthalen-1-yl)-8-oxo-1,5,6,8-tetrahydro-2H-1,5-methanopyrido[1,2-a][1,5]diazocine-3(4H)-carbothioamide
7	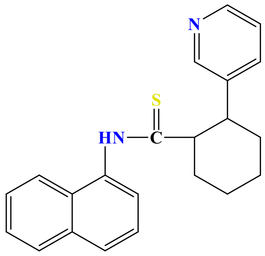	N-(naphthalen-1-yl)-2-(pyridin-3-yl)cyclohexane-1-carbothioamide

Table S2. Conformers of **1-7** compounds and their MMFF94 steric energies

Compound	Conformers and their MMFF94 calculated energies
1	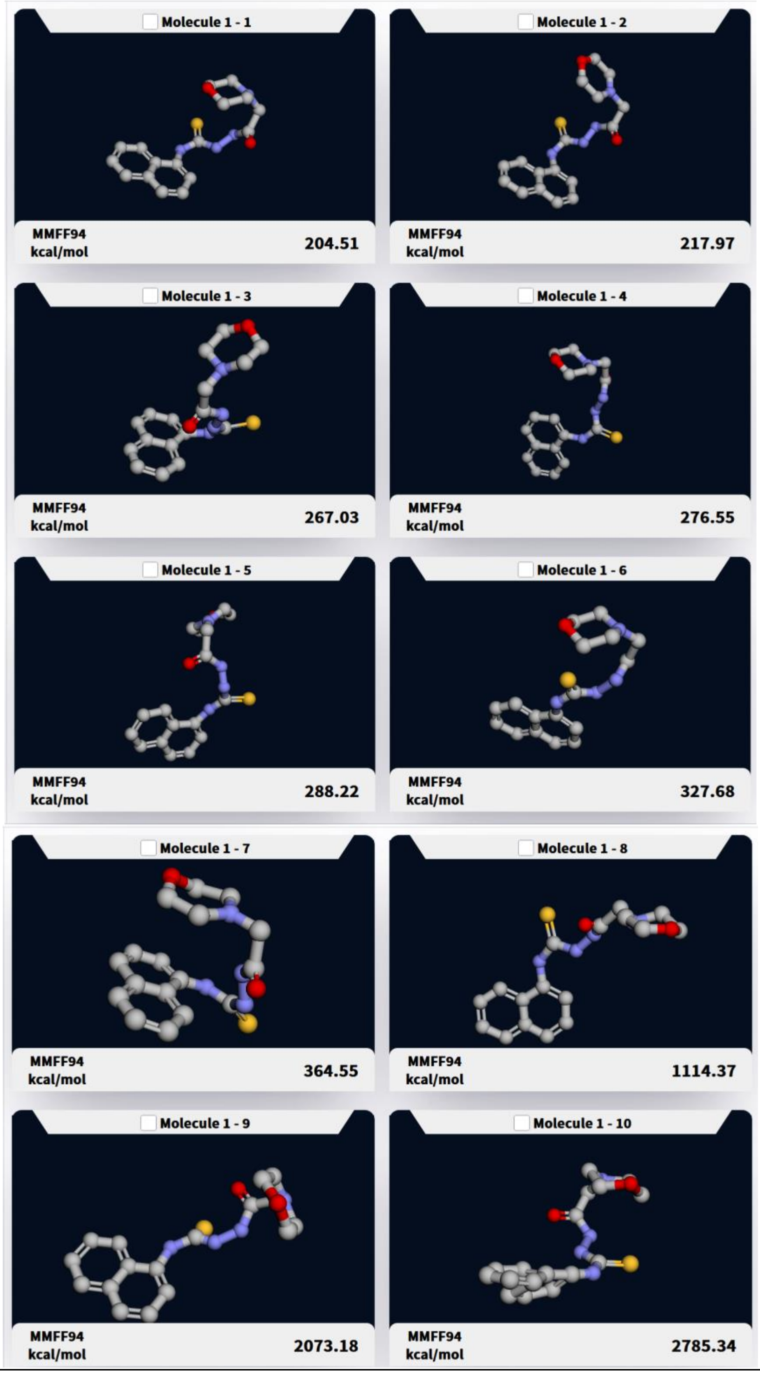

<table>
<tr><td>2</td><td>
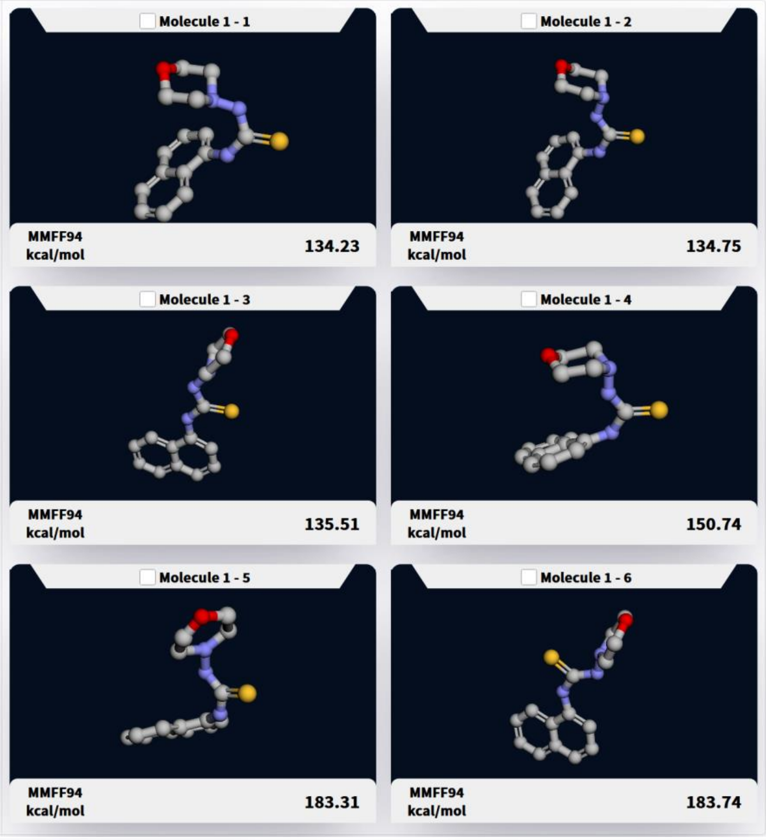

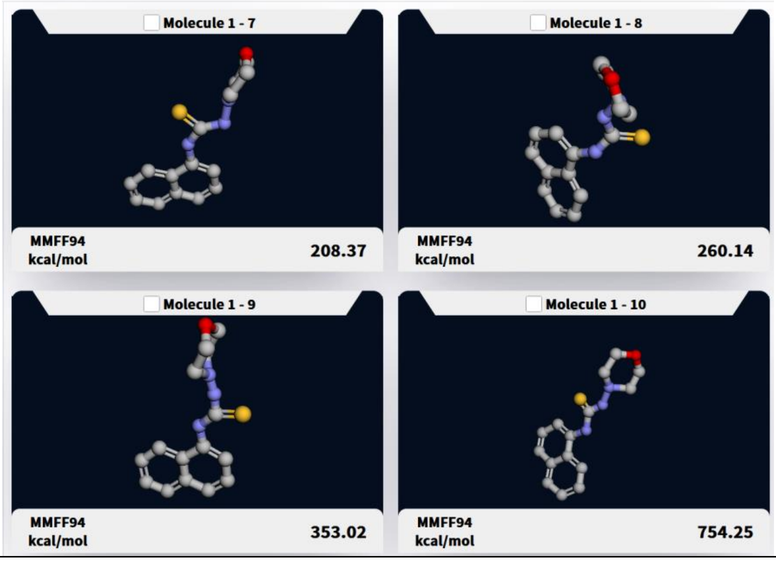

</td></tr>
</table>

3		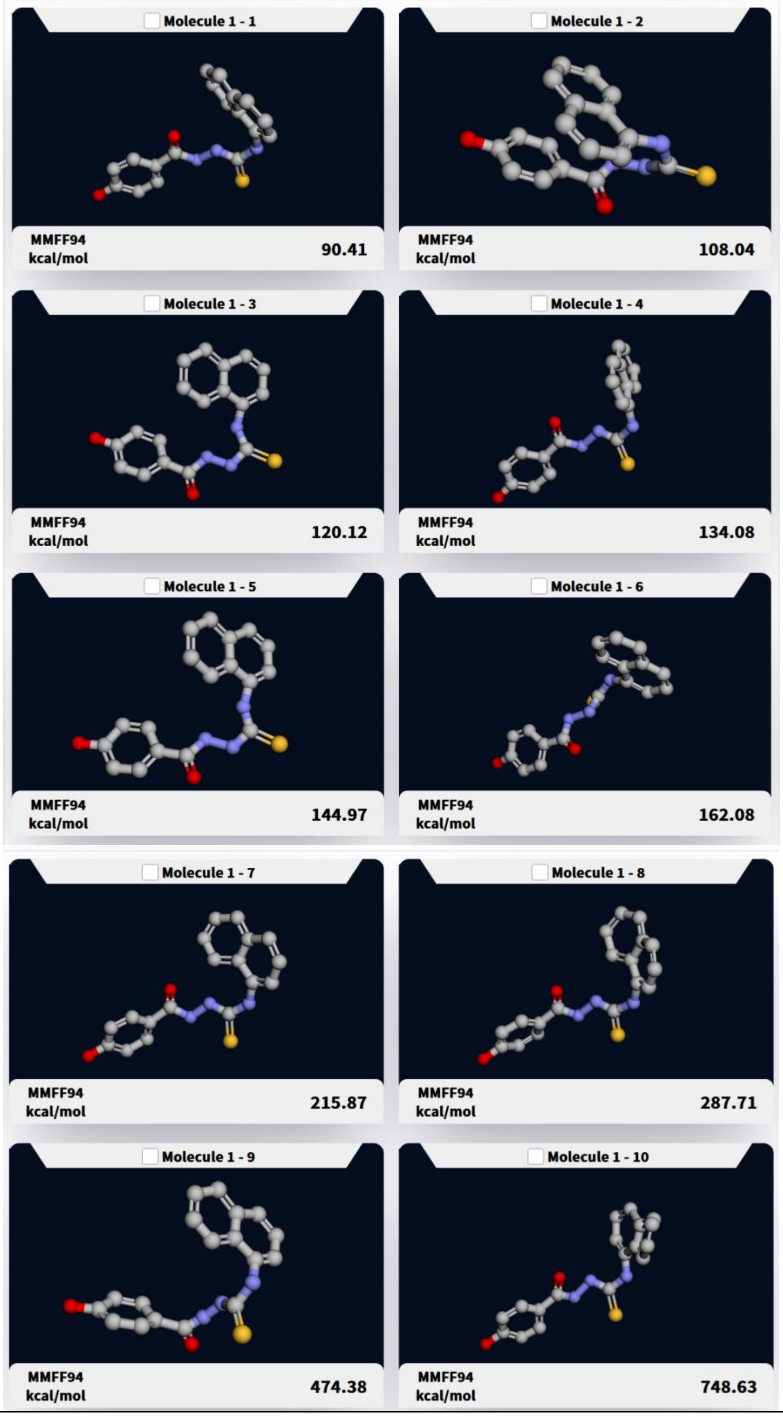Molecule 1 - 1<br>MMFF94 kcal/mol 90.41<br>Molecule 1 - 2<br>MMFF94 kcal/mol 108.04<br>Molecule 1 - 3<br>MMFF94 kcal/mol 120.12<br>Molecule 1 - 4<br>MMFF94 kcal/mol 134.08<br>Molecule 1 - 5<br>MMFF94 kcal/mol 144.97<br>Molecule 1 - 6<br>MMFF94 kcal/mol 162.08<br>Molecule 1 - 7<br>MMFF94 kcal/mol 215.87<br>Molecule 1 - 8<br>MMFF94 kcal/mol 287.71<br>Molecule 1 - 9<br>MMFF94 kcal/mol 474.38<br>Molecule 1 - 10<br>MMFF94 kcal/mol 748.63

4		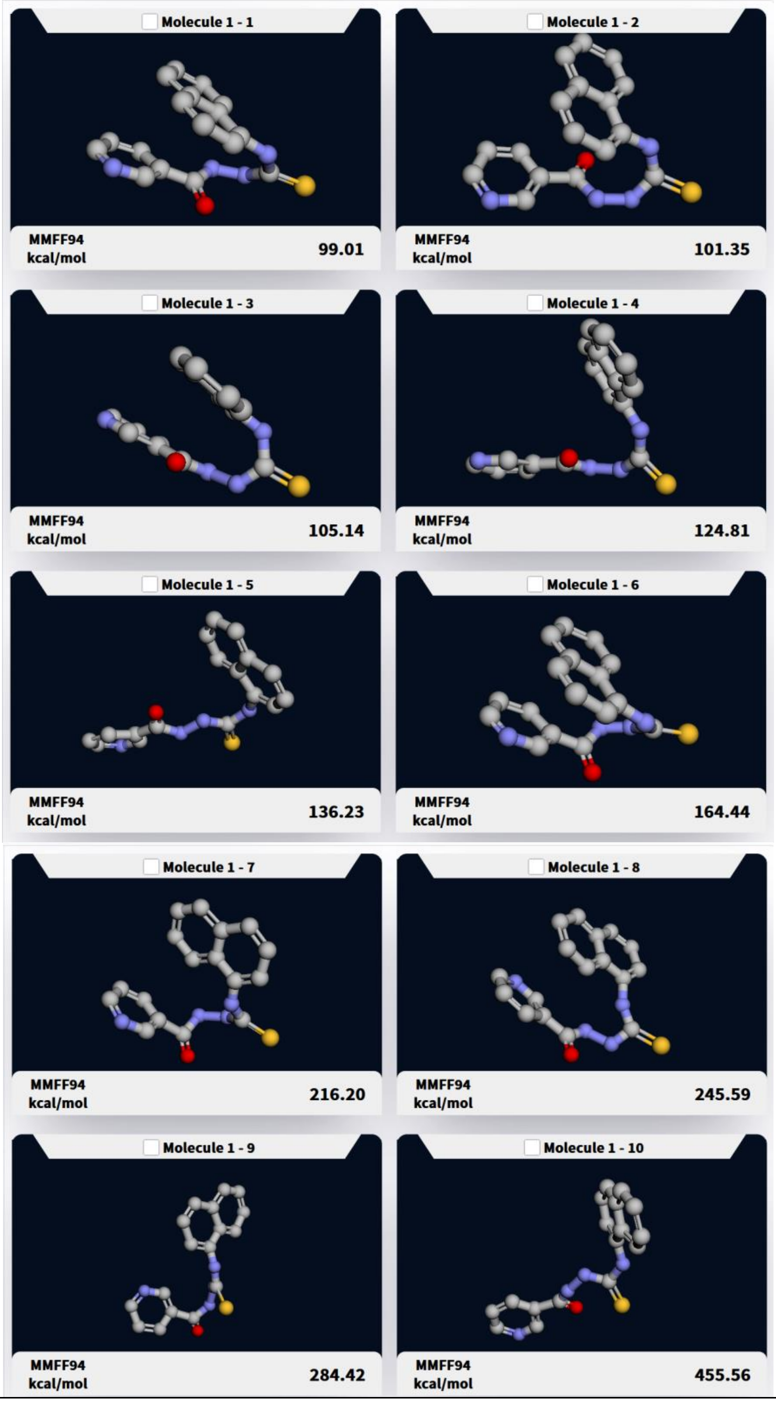

Molecule 1 - 1
MMFF94 kcal/mol 99.01
Molecule 1 - 2
MMFF94 kcal/mol 101.35
Molecule 1 - 3
MMFF94 kcal/mol 105.14
Molecule 1 - 4
MMFF94 kcal/mol 124.81
Molecule 1 - 5
MMFF94 kcal/mol 136.23
Molecule 1 - 6
MMFF94 kcal/mol 164.44
Molecule 1 - 7
MMFF94 kcal/mol 216.20
Molecule 1 - 8
MMFF94 kcal/mol 245.59
Molecule 1 - 9
MMFF94 kcal/mol 284.42
Molecule 1 - 10
MMFF94 kcal/mol 455.56

5		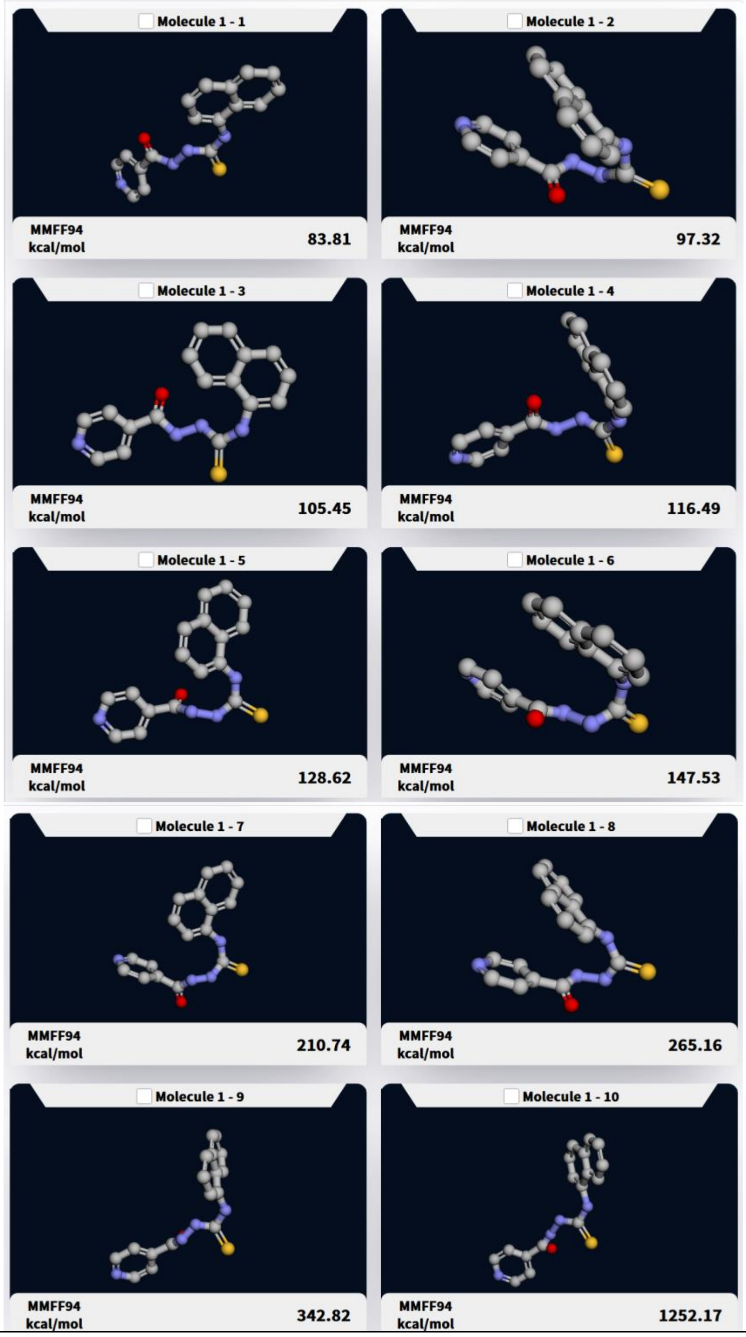

Molecule 1 - 1
MMFF94 kcal/mol 83.81
Molecule 1 - 2
MMFF94 kcal/mol 97.32
Molecule 1 - 3
MMFF94 kcal/mol 105.45
Molecule 1 - 4
MMFF94 kcal/mol 116.49
Molecule 1 - 5
MMFF94 kcal/mol 128.62
Molecule 1 - 6
MMFF94 kcal/mol 147.53
Molecule 1 - 7
MMFF94 kcal/mol 210.74
Molecule 1 - 8
MMFF94 kcal/mol 265.16
Molecule 1 - 9
MMFF94 kcal/mol 342.82
Molecule 1 - 10
MMFF94 kcal/mol 1252.17

<table>
<tr>
<td>6</td>
<td>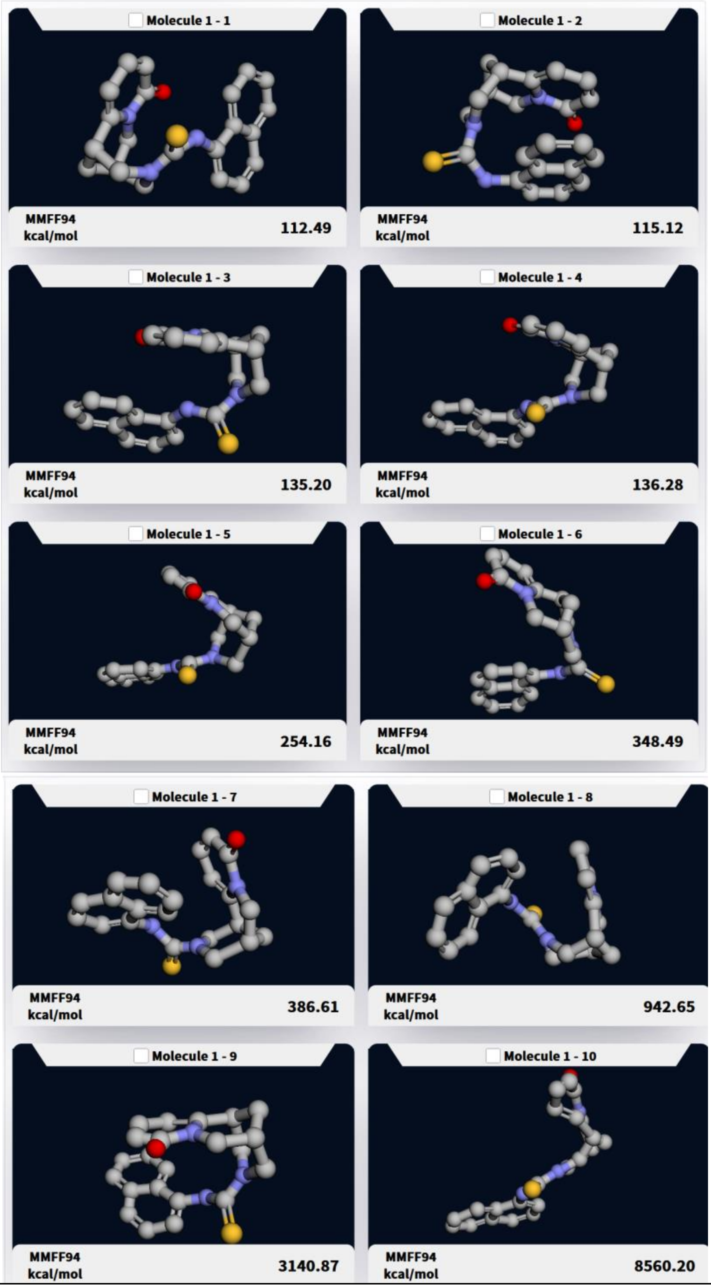

</td>
</tr>
</table>

<table>
<tr>
<td>7</td>
<td>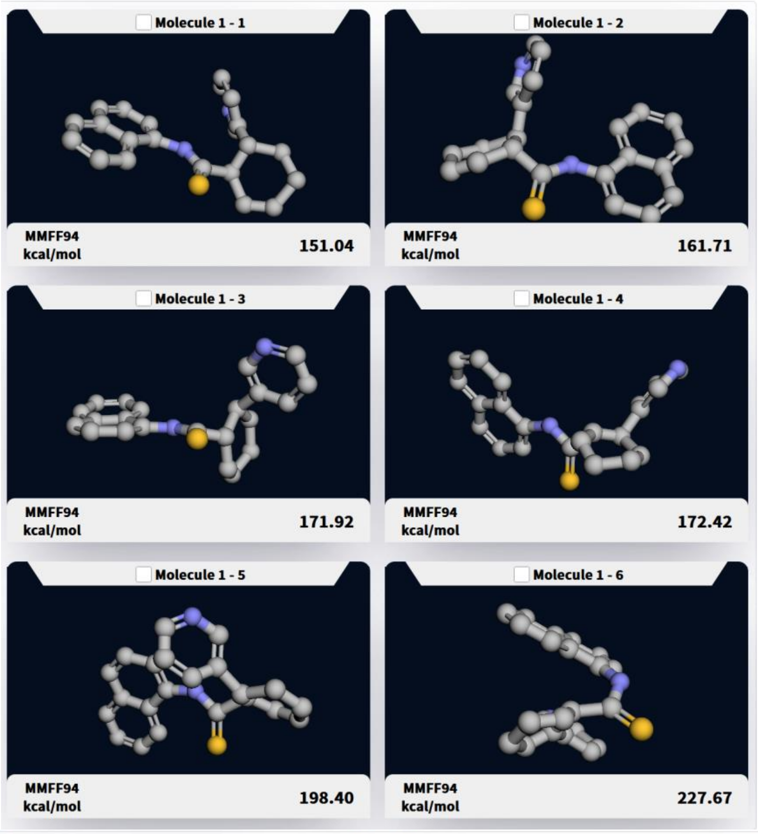

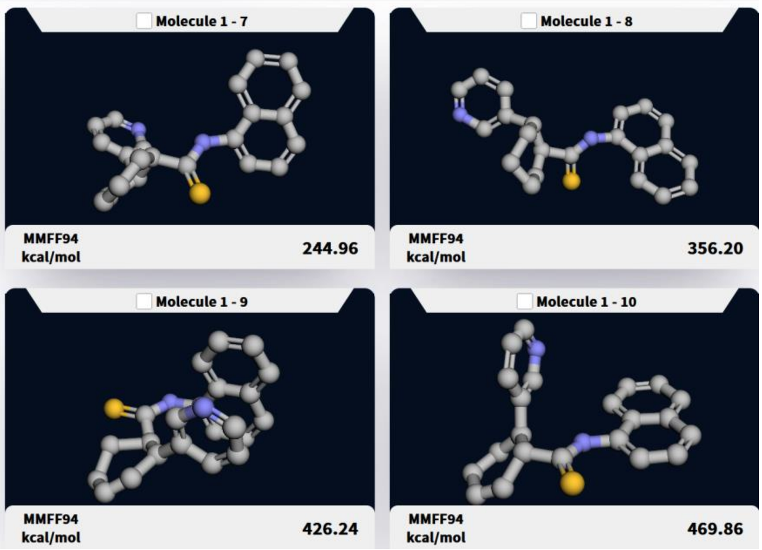
</td>
</tr>
</table>

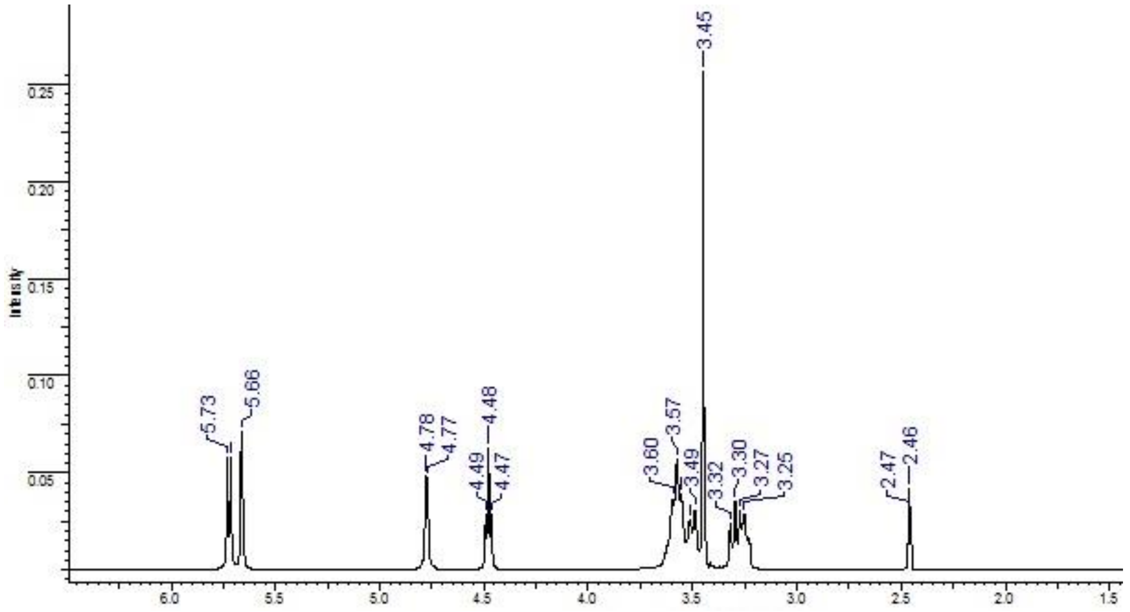

Figure S4. ^{1}H NMR spectrum of β-CD (DMSO)

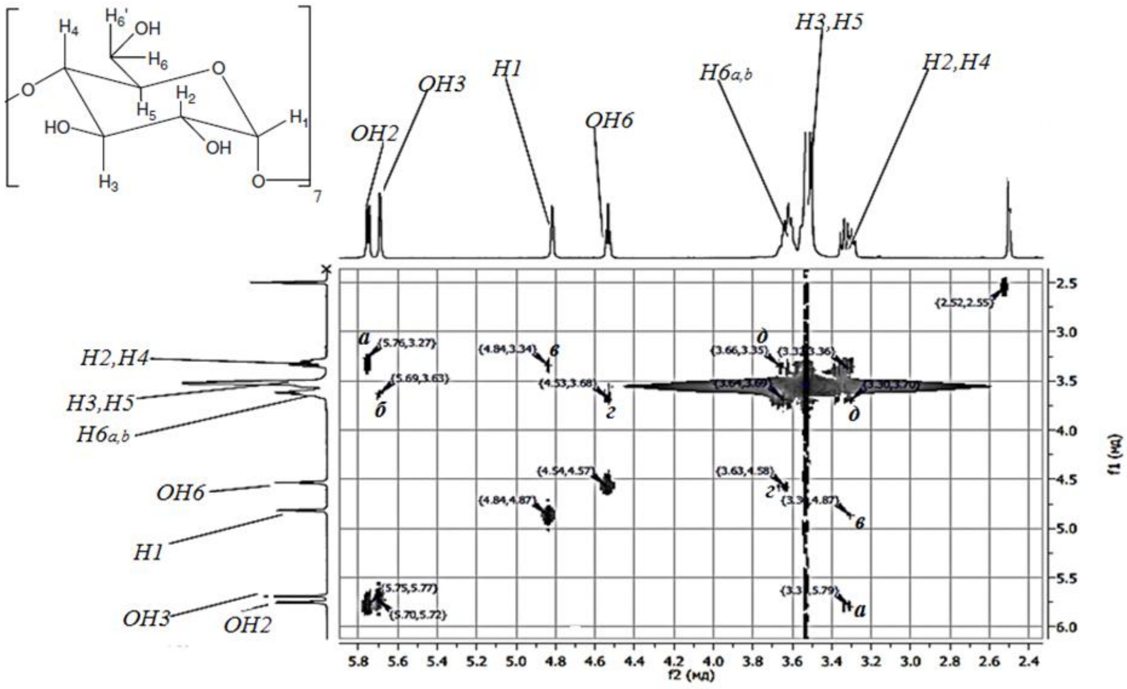

Figure S5. COSY (^{1}H-^{1}H) NMR spectrum of β-CD (DMSO)

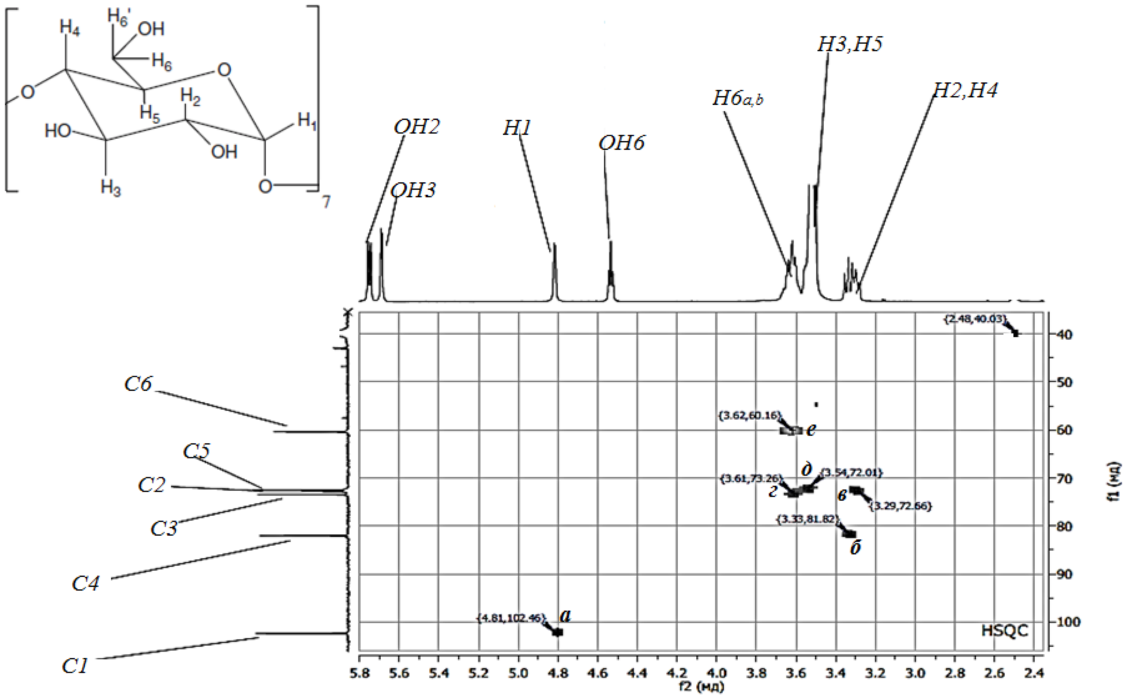

Figure S6. HSQC (^1H-^{13}C) NMR spectrum of β-CD (DMSO)

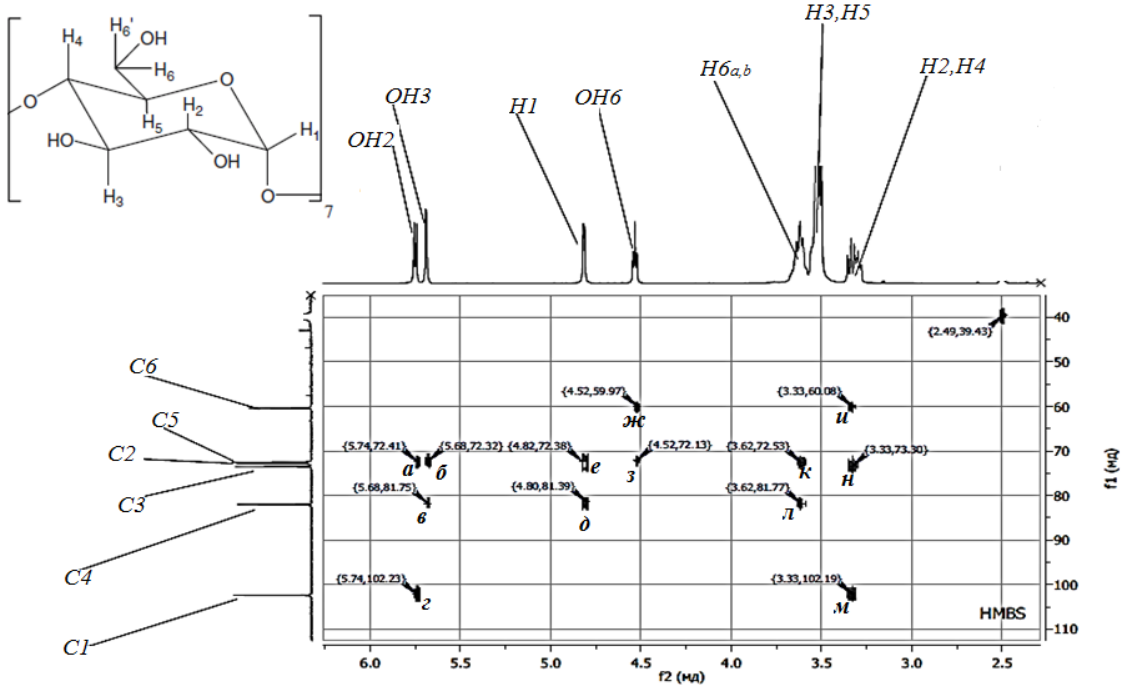

Figure S7. HMBC (^1H-^{13}C) NMR spectrum of β-CD (DMSO)

Analysis of β-CD NMR Spectra

The ^1H NMR spectrum of β-CD, obtained in deuterated dimethyl sulfoxide (DMSO), is characterized by six groups of signals in the range of 3.23-3.32; 3.45-3.53; 3.56-3.60; 4.47-4.49; 4.77-4.78; and 5.66-5.73 ppm (Fig. S4). The lowest-field doublet signal in the range of 5.71-5.73 ppm with a splitting of 4 Hz belongs to the proton of the hydroxyl group at the C-2 atom (OH-2). The proton of the OH group of the adjacent atom (OH-3), located in the internal cavity of the CD molecule, also resonates in the downfield region (δ = 5.66 ppm, doublet). The doublet signal in the region of 4.77-4.78 ppm corresponds to the H-1 proton. The location of this proton in a weaker field compared to the protons of other CH groups is due to the influence of the oxygen atom. The hydroxyl group OH-6 resonates, splitting into a triplet

centered at 4.48 ppm. In the strong field region (3.56-3.60 ppm), an accumulation of signals of the H-6 a, b methylene group is observed. The high-intensity signal at 3.45 ppm corresponds to the H-3 and H-5 protons of the glucopyranose unit. In the range from 3.23 to 3.32 ppm, the methine protons H-2 and H-4 appear.

The carbon spectrum of β-CD consists of five signals from the ^{13}C nuclei of the elementary unit. The upfield signal is due to the C-6 carbon atom (δ = 60.41 ppm). The signals at 72.49, 72.85, and 73.51 ppm are due to the C-5, C-2, and C-3 atoms, respectively. Downfield signals (82.02 and 102.41 ppm) are due to the carbon atoms (C-4 and C-1, respectively), directly linked to the adjacent glucopyranose unit via an oxygen bridge.To obtain more complete and accurate information on homo- (^1H-^1H) and heteronuclear (^1H-^{13}C) correlations, two-dimensional NMR spectra were acquired and interpreted in COSY, HSQC, and HMBC formats. The interactions between the ^1H nuclei can be determined using the COSY spectrum shown in Fig. S5. The presence of spin interaction, mediated through 3 bonds, between the proton at the carbon atom of the glucopyranose unit and the proton of the corresponding hydroxyl group is indicated by crosspeaks with coordinates (3.30; 5.79) H2–OH2; (3.63; 4.58) H6–OH6. The correlation with coordinates (3.34; 4.87) corresponds to interaction through 3 bonds between the H-1 and H-2 protons. A similar relationship is observed for the H2–H3, H3–H4, and H4–H5 protons. The interactions of the ^1H–^{13}C nuclei, mediated through 1, 2, and 3 bonds, can be traced using the HMBC (Fig. S7) and HSQC spectra (Fig. S6). Heteronuclear interactions through 1 bond (Fig. S6) are characteristic of the nuclei H1–C1 (4.81; 102.46), H2–C2 (3.29; 72.66), H3–C3 (3.61; 73.26), H4–C4 (3.33; 81.82), H5–C5 (3.54; 72.01), H6–C6 (3.62; 60.16). The following interactions are characteristic of the hydroxyl proton at the C2 carbon atom: OH2-C2 and OH2-C3, determined by fragment a (5.74; 72.41), as well as OH2-C1 - the peak with coordinates (5.74; 102.23). It should be noted that the first type of interaction occurs through 2 bonds, and the next two - through 3. The hydroxyl group at the third carbon atom (OH-3) also interacts with neighboring 13C nuclei through two (OH3-C3) and three bonds (OH3-C2; OH3-C4), as evidenced by peaks b (5.68; 72.32) and c (5.68; 81.75). Peak e (4.82; 72.38) corresponds to H1-C2 and H1-C3 interactions, realized through 2 and 3 bonds, respectively. Also, this proton is characterized by a correlation with the carbon atom C4 d (4.80; 81.39). The hydroxyl group OH-6 is determined by the peaks (4.52; 59.97) and (4.52; 72.13), which correspond to the bonds OH6–C6 (through two bonds) and OH6–C5 (through three bonds). The peak (3.62; 72.53) indicates the interactions of the proton-carbon nuclei H6–C5 (through two bonds). Also, this proton is characterized by correlations with the C-4 atom (3.62; 81.77). Protons H-2 and H-4 interact with carbon atoms C3 and C5, as confirmed by the cross peak (3.33; 73.30). For H-2, a correlation with the C-1 atom at the point (3.33; 102.19) was also determined.

Table S3. Chemical shifts of ^1H and ^{13}C cyclodextrin nuclei in the free state (δ0) and as part of the inclusion complex with (δ)

Atom number	Group	δ_0, ppm.		δ, ppm.		$\Delta\delta = \delta - \delta_0$	
		^1H	^{13}C	^1H	^{13}C	^1H	^{13}C
β -Cyclodextrin inclusion complex with **1**							
1	CH	4.75 с	102.82	4.74	102.42	-0.01	-0.40
2	CH	3.23 м	72.86	3.22	72.91	-0.01	0.05
3	CH	3.60 м	73.64	3.57	73.37	-0.03	-0.27
4	CH	3.27 м	81.88	3.24	82.11	-0.03	-0.23
5	CH	3.50 м	72.51	3.51	73.71	0.01	-0.20
6	CH	3.60 м	60.42	3.57	60.37	-0.03	-0.05
β -Cyclodextrin inclusion complex with **2**							
1	CH	4.77 с	102.85	4.76	102.55	-0.01	-0.30
2	CH	3.24 м	72.87	3.23	72.90	-0.01	0.03
3	CH	3.60 м	73.64	3.56	73.37	-0.04	-0.28
4	CH	3.28 м	81.98	3.25	82.11	-0.03	-0.87
5	CH	3.49 м	72.50	3.50	73.21	0.01	-0.29
6	CH	3.60 м	60.42	3.57	60.37	-0.03	-0.05
β -Cyclodextrin inclusion complex with **3**							
1	CH	4.75 с	102.87	4.74	102.41	-0.01	-0.46
2	CH	3.24 м	72.87	3.24	72.84	0	-0.03
3	CH	3.63 м	73.64	3.59	73.55	-0.04	-0.09
4	CH	3.28 м	81.98	3.25	81.99	-0.03	0.01
5	CH	3.49 м	72.50	3.51	72.51	0.02	-0.01
6	CH	3.60 м	60.42	3.56	60.43	-0.04	0.01
β -Cyclodextrin inclusion complex with **4**							
1	CH	4.83 с	102.20	4.82	102.31	-0.01	0.10

2	CH	3.25 м	72.69	3.26	73.04	0.01	0.35
3	CH	3.60 м	73.43	3.57	73.07	-0.03	-0.38
4	CH	3.28 м	81.46	3.29	81.75	0.01	0.29
5	CH	3.38 м	73.11	3.42	73.27	0.04	-0.16
6	CH	3.58 м	60.53	3.55	60.34	-0.19	-0.19
β -Cyclodextrin inclusion complex with **5**							
1	CH	4.77 с	102.87	4.76	102.41	-0.01	-0.46
2	CH	3.24 м	72.87	3.25	72.84	0.01	-0.03
3	CH	3.65 м	73.64	3.62	73.55	-0.03	-0.09
4	CH	3.28 м	81.98	3.25	81.99	-0.03	0.01
5	CH	3.50 м	72.50	3.54	72.51	0.04	-0.01
6	CH	3.60 м	60.42	3.56	60.43	-0.04	0.01
β -Cyclodextrin inclusion complex with **6**							
1	CH	4.71 с	102.87	4.70	102.41	-0.01	-0.46
2	CH	3.24 м	72.87	3.25	72.84	0.01	-0.03
3	CH	3.67 м	73.64	3.62	73.55	-0.05	-0.09
4	CH	3.30 м	81.98	3.29	81.99	-0.01	0.01
5	CH	3.47 м	72.50	3.52	72.51	0.05	-0.01
6	CH	3.60 м	60.42	3.56	60.43	-0.04	0.01
β -Cyclodextrin inclusion complex with **7**							
1	CH	4.76 с	102.87	4.75	102.37	-0.01	-0.5
2	CH	3.23 м	72.86	3.22	72.91	-0.01	0.05
3	CH	3.62 м	73.63	3.56	73.37	-0.06	-0.26
4	CH	3.27 м	81.98	3.26	82.11	-0.01	-0.87
5	CH	3.53 м	72.50	3.57	73.21	0.04	-0.29
6	CH	3.60 м	60.42	3.57	60.37	-0.03	-0.05
7	CH	4.77 с	102.87	4.75	102.37	-0.02	-0.5

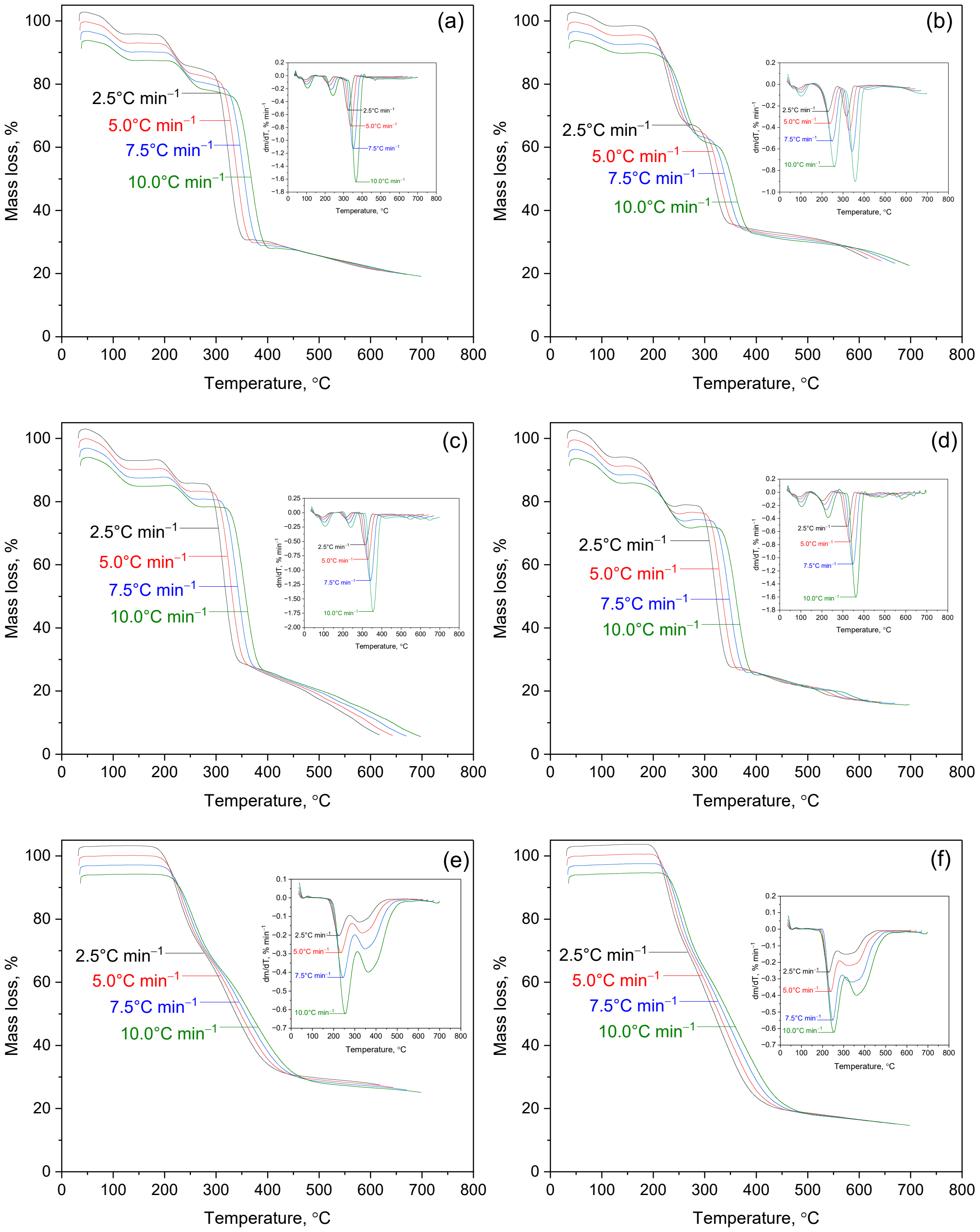

Figure S8. Thermogravimetric (TGA) and differential thermogravimetric (DTG) curves of inclusion complexes with β-cyclodextrins, recorded at heating rates of 2.5, 5.0, 7.5, and 10.0°C/min: (a) Inclusion complex of 2-(morpholinoacetyl)-N-(naphthalen-1-yl)hydrazine-1-carbothioamide with β-CD; (b)-Inclusion complex of 1-morpholino-3-(naphthalen-1-yl)thiourea with β-CD; (c) - Inclusion complex of 2-(4-hydroxybenzoyl)-N-(naphthalen-1-yl)-hydrazino-1-carbothioamide with β-CD;
(d) - Inclusion complex of N-(naphthalen-1-yl)-anabazine-1-carbothioamide with β-CD;
(e) - Inclusion complex of N-(naphthalene-1-yl)-2-nicotinoylhydrazono-1-carbothioamide with β-CD; (f) - Inclusion complex of N-(naphthalene-1-yl) 2-isonicotinoylhydrazono-1-carbothioamide with β-CD.

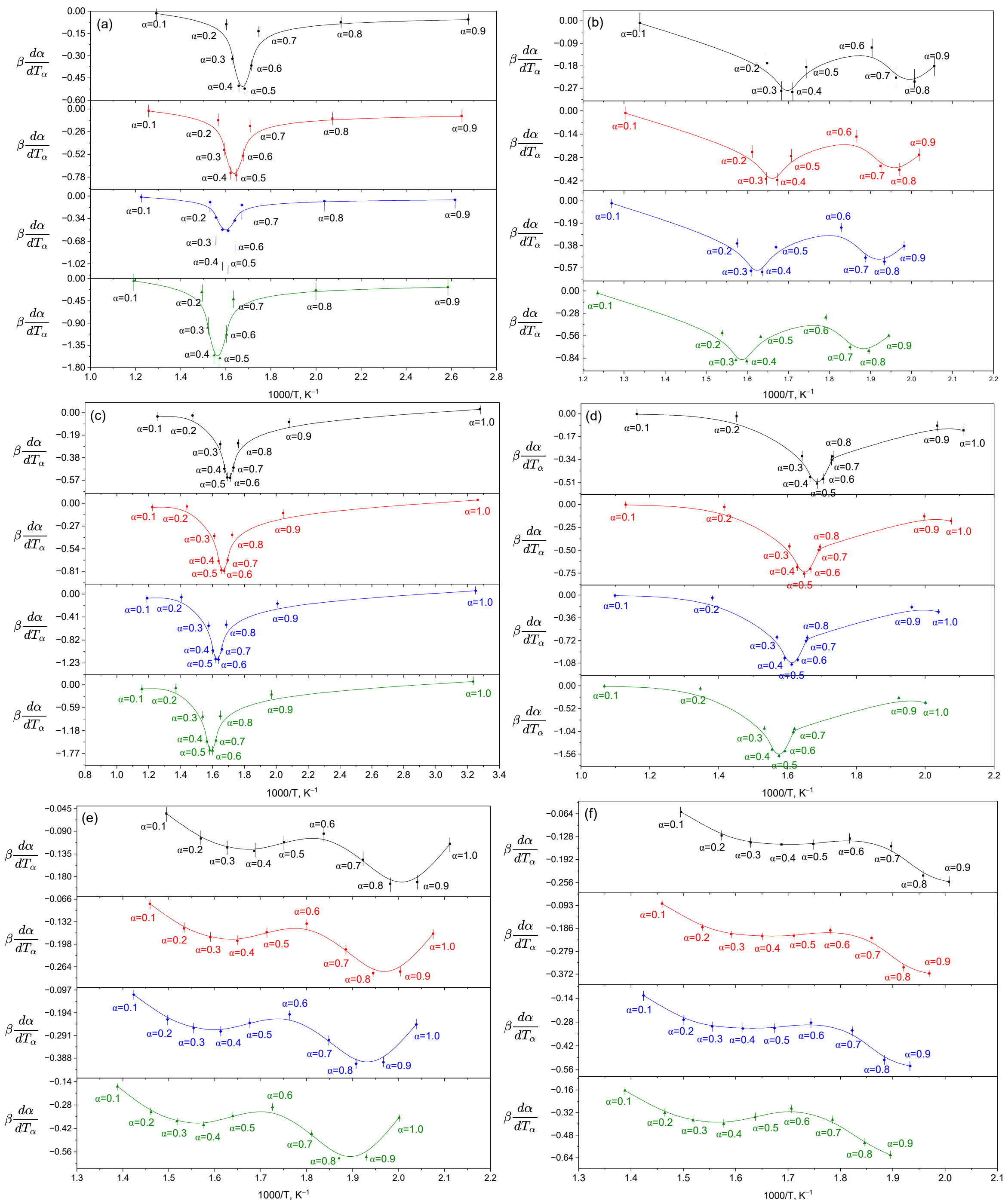

Figure S9. Dependences of log β on $1/T$, constructed according to the Ozawa-Flynn-Wall method for calculating the activation energy of thermal decomposition of inclusion complexes with β-cyclodextrins at different degrees of transformation (α=0.1–0.9). The calculations were performed based on TGA data obtained at heating rates of 2.5, 5.0, 7.5, and 10.0 °C/min: (a) - Inclusion complex of 2-(morpholinoacetyl)-N-(naphthalen-1-yl)hydrazine-1-carbothioamide with β-CD; (b) - Inclusion complex of 1-morpholino-3-(naphthalen-1-yl)thiourea with β-CD; (c) - Inclusion complex of 2-(4-hydroxyphenyl)-N-(naphthalen-1-yl)-hydrazino-1-carbothioamide with β-CD; (d) - Inclusion complex of N-(naphthalen-1-yl)-anabazine-1-carbothioamide with β-CD; (e) - Inclusion complex of N-(naphthalene-1-yl)-2-nicotinoylhydrazono-1-carbothioamide with β-CD; (f) - Inclusion complex of N-(naphthalene-1-yl) 2-isonicotinoylhydrazono-1-carbothioamide with β-CD.

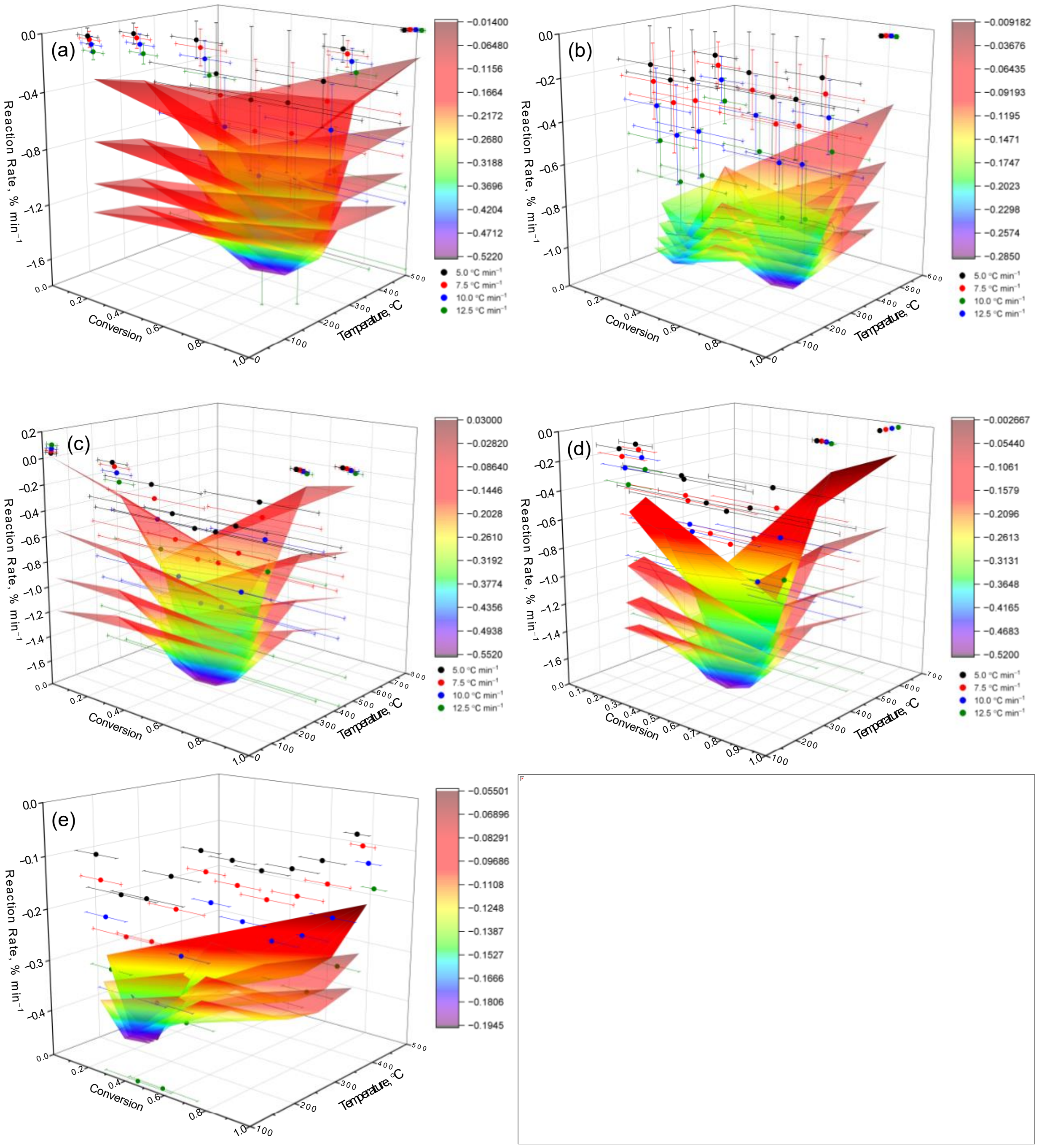

Figure S10. Three-dimensional dependences of $d\alpha/dT$ on temperature T and degree of conversion α for inclusion complexes with β-cyclodextrins, calculated at various rates of 5.0; 7.5; 10.0, and 12.5°C min^{-1}: (a) - Inclusion complex of 2-(morpholinoacetyl)-N-(naphthalen-1-yl)hydrazine-1-carbothioamide with β-CD; (b) - Inclusion complex of 1-morpholino-3-(naphthalen-1-yl)thiourea with β-CD; (c) - Inclusion complex of 2-(4-hydroxyphenyl)-N-(naphthalen-1-yl)-hydrazino-1-carbothioamide with β-CD; (d) - Inclusion complex of N-(naphthalen-1-yl)-anabazine-1-carbothioamide with β-CD; (e) - Inclusion complex of N-(naphthalene-1-yl)-2-nicotinoylhydrazono-1-carbothioamide with β-CD; (f) - Inclusion complex of N-(naphthalene-1-yl) 2-isonicotinoylhydrazono-1-carbothioamide with β-CD.

Table S4. Effect of samples **1-7** on blood viscosity (mPa*s) at different spindle rotation speeds on an *in vitro* blood hyperviscosity model (incubation at 43°C)

The indicator under study	Blood viscosity (mPa*s) at different spindle rotation speeds, rpm							
	2	4	6	8	12	20	40	60
**N-(naphthalene-1-yl)- 2-isonicothionyl-hydrazino-1carbothioamide**								
Initial viscosity, n=2	3,74±0,04	3,16±0,02	2,59±0,03	1,88±0,03	1,5±0,06	1,08±0,03	0,99±0,01	0,93±0,01
Blood viscosity after 1 hour in the control, n=4	5,62±0,17<br>p1=0,004	4,2±0,07<br>p1=0,0012	3,17±0,13<br>p1=0,0758	2,68±0,05<br>p1=0,0013	2,25±0,05<br>p1=0,0020	1,97±0,02<br>p1=0,00002	1,28±0,02<br>p1=0,029	1,08±0,02<br>p1=0,0196
Blood viscosity after 1 hour of testing with compound, n=4	6,48±0,14<br>p1=0,0004<br>p2=0,0193	4,56±0,06<br>p1=0,0002<br>p2=0,0185	3,58±0,04<br>p1=0,0001<br>p2=0,0508	2,93±0,02<br>p1=0,0001<br>p2=0,0207	2,4±0,03<br>p1=0,0001<br>p2=0,0625	2,1±0,03<br>p1=0,00001<br>p2=0,0163	1,5±0,03<br>p1=0,0004<br>p2=0,0021	1,2±0,01<br>p1=0,00008<br>p2=0,0072
**N-(naphthalene-1-yl)-2-nicothionyl- hydrazino-1-carbothioamide**								
Initial viscosity, n=2	4,72±0,16	3,47±0,29	2,89±0,24	2,47±0,02	1,92±0,16	1,53±0,21	0,64±0,09	0,23±0,08
Blood viscosity after 1 hour in the control, n=4	5,56±0,28<br>p1=0,1830	4,55±0,14<br>p1=0,0308	3,68±0,09<br>p1=0,0361	3,2±0,03<br>p1=0,0001	2,51±0,16<br>p1=0,1301	2,27±0,17<br>p1=0,1079	1,41±0,12<br>p1=0,0293	0,97±0,14<br>p1=0,0481
Blood viscosity after 1 hour of testing with compound, n=4	7,15±0,04<br>p1=0,0001<br>p2=0,0036	5,31±0,06<br>p1=0,0017<br>p2=0,0060	4,24±0,02<br>p1=0,0019<br>p2=0,0031	3,55±0,03<br>p1=0,0001<br>p2=0,0005	3,19±0,01<br>p1=0,0004<br>p2=0,0125	2,39±0,08<br>p1=0,0158<br>p2=0,6103	2,01±0,07<br>p1=0,0006<br>p2=0,0126	1,4±0,04<br>p1=0,0002<br>p2=0,0626
**N-2-(2-morpholinoacetyl) -(naphthalene-1-yl)-hydrazino-1-carbotioamide**								
Initial viscosity, n=2	4,57±0,38	3,62±0,30	2,73±0,32	2,33±0,25	2,02±0,09	1,53±0,04	0,66±0,36	0,45±0,27
Blood viscosity after 1 hour in the control, n=4	8,34±0,08<br>p1=0,0002	5,67±0,03<br>p1=0,0009	4,27±0,03<br>p1=0,0033	3,7±0,05<br>p1=0,0029	2,92±0,04<br>p1=0,0005	2,23±0,03<br>p1=0,0002	1,72±0,03<br>p1=0,0173	1,28±0,02<br>p1=0,0155
Blood viscosity after 1 hour of testing with compound, n=4	7,60±0,24<br>p1=0,0043<br>p2=0,0508	5,31±0,05<br>p1=0,0023<br>p2=0,0018	4,11±0,03<br>p1=0,0051<br>p2=0,0227	3,07±0,25<br>p1=0,2080<br>p2=0,0891	2,44±0,17<br>p1=0,2434<br>p2=0,0588	2,09±0,04<br>p1=0,0031<br>p2=0,0565	1,32±0,04<br>p1=0,0727<br>p2=0,0006	1,09±0,02<br>p1=0,0359<br>p2=0,0008
**N-2-4(4-hydroxybenzoyl)-(naphthalene-1-yl)-hydrazino -1-carbotioamide**								
Initial viscosity, n=2	4,9±0,63	3,54±0,24	2,98±0,10	2,47±0,02	2,07±0,04	1,52±0,12	1,13±0,09	0,99±0,10
Blood viscosity after 1 hour in the control, n=4	6,64±0,19<br>p1=0,0408	4,6±0,30<br>p1=0,1404	3,63±0,24<br>p1=0,2209	3,01±0,14<br>p1=0,1071	2,43±0,11<br>p1=0,1546	2,04±0,11<br>p1=0,0747	1,41±0,11<br>p1=0,2442	1,18±0,06<br>p1=0,2159
Blood viscosity after 1 hour of testing with compound, n=4	6,29±0,23<br>p1=0,0929<br>p2=0,3657	4,37±0,19<br>p1=0,1044<br>p2=0,6127	3,47±0,14<br>p1=0,1499<br>p2=0,6610	2,96±0,13<br>p1=0,1149<br>p2=0,8313	2,47±0,10<br>p1=0,0969<br>p2=0,8554	1,97±0,09<br>p1=0,0662<br>p2=0,6951	1,28±0,08<br>p1=0,4175<br>p2=0,4615	1,05±0,06<br>p1=0,6673<br>p2=0,2688
**1-morpholino-3-(naphthalene-1-yl)-thiourea**								
Initial viscosity, n=2	6,2±0,07	4,67±0,51	3,46±0,14	2,85±0,08	2,39±0,04	1,91±0,03	1,27±0,02	0,64±0,24
Blood viscosity after 1 hour in the control, n=4	9,47±0,62<br>p1=0,0458	7,46±0,14<br>p1=0,0038	6,08±0,05<br>p1=0,0001	4,65±0,08<br>p1=0,0003	3,78±0,13<br>p1=0,0041	3,41±0,12<br>p1=0,00232	2,23±0,02<br>p1=0,00003	2,1±0,03<br>p1=0,0015
Blood viscosity after 1 hour of testing with	7,5±0,33<br>p1=0,1011<br>p2=0,0631	6,21±0,03<br>p1=0,0161<br>p2=0,0004	4.32±0,12<br>p1=0,0220<br>p2=0,00003	3,55±0,11<br>p1=0,0302<br>p2=0,0006	2,93±0,04<br>p1=0,0025<br>p2=0,0022	2,38±0,09<br>p1=0,0551<br>p2=0,0013	1,49±0,01<br>p1=0,0008<br>p2=0,00005	1,32±0,02<br>p1=0,0228<br>p2=0,00001

compound, n=4								
**1N-(Naphthalene-1-yl)cytisino-1-carbotioamide**								
Initial viscosity, n=2	5,59±0,93	4,03±0,41	3,12±0,81	2,67±0,84	1,91±0,31	1,14±0,03	0,41±0,05	0,3±0,01
Blood viscosity after 1 hour in the control, n=4	8,51±0,83 p1=0,1566	6,43±0,81 p1=0,1897	5,19±0,42 p1=0,1012	3,95±0,29 p1=0,1929	2,9±0,18 p1=0,07020	2,13±0,08 p1=0,0025	1,39±0,02 p1=0,00005	1,18±0,01 p1=0,0003
Blood viscosity after 1 hour of testing with compound, n=4	9,83±0,31 p1=0,0090 p2=0,2721	7,71±0,75 p1=0,0593 p2=0,3815	5,81±0,23 p1=0,0224 p2=0,3337	4,39±0,15 p1=0,0664 p2=0,3217	3,57±0,02 p1=0,0022 p2=0,0225	2,36±0,04 p1=0,0001 p2=0,867	1,6±0,04 p1=0,0001 p2=0,0091	1,27±0,01 p1=0,00042 p2=0,0044
**N-(Naphthalene-1-yl)anabazino-1-carbotioamide**								
Initial viscosity, n=2	4,79±0,04	4,14±0,38	3,41±0,18	2,68±0,22	2,22±0,19	1,71±0,20	1,41±0,07	0,9±0,09
Blood viscosity after 1 hour in the control, n=4	9,1±0,33 p1=0,0021	7,02±0,17 p1=0,0023	5,72±0,29 p1=0,0143	4,28±0,23 p1=0,0231	3,07±0,10 p1=0,0205	2,38±0,11 p1=0,0555	1,87±0,10 p1=0,0662	1,63±0,19 p1=0,1131
Blood viscosity after 1 hour of testing with compound, n=4	8,16±0,42 p1=0,0119 p2=0,2022	6±0,10 p1=0,0053 p2=0,0051	4,39±0,10 p1=0,0135 p2=0,0130	3,12±0,24 p1=0,4033 p2=0,0280	2,35±0,10 p1=0,6063 p2=0,0059	2,06±0,08 p1=0,1689 p2=0,0976	1,52±0,05 p1=0,3394 p2=0,0401	1,27±0,08 p1=0,0683 p2=0,2145
Note:<br>n – number of samples in the group;<br>p – significance level;<br>p1<0.05 – statistically significant differences compared to the initial values;<br>p2<0.05 – statistically significant differences compared to the corresponding values in the control samples								
